# A common genetic variation in *GZMB* may associate with cancer risk in patients with Lynch syndrome

**DOI:** 10.3389/fonc.2023.1005066

**Published:** 2023-02-20

**Authors:** Vince Kornél Grolmusz, Petra Nagy, István Likó, Henriett Butz, Tímea Pócza, Anikó Bozsik, János Papp, Edit Oláh, Attila Patócs

**Affiliations:** ^1^ Department of Molecular Genetics, National Institute of Oncology, Budapest, Hungary; ^2^ Hereditary Cancers Research Group, Eötvös Loránd Research Network – Semmelweis University, Budapest, Hungary; ^3^ Department of Laboratory Medicine, Semmelweis University, Budapest, Hungary; ^4^ National Tumorbiology Laboratory, National Institute of Oncology, Budapest, Hungary; ^5^ National Oncology Biobank Center, National Institute of Oncology, Budapest, Hungary

**Keywords:** granzyme B, Lynch syndrome, immunotherapy, microsatellite instability, colorectal cancer, mismatch repair deficiency, cancer neoantigens, immune infiltration

## Abstract

Lynch syndrome (LS), also known as hereditary nonpolyposis colorectal cancer syndrome (HNPCC) is a common genetic predisposition to cancer due to germline mutations in genes affecting DNA mismatch repair. Due to mismatch repair deficiency, developing tumors are characterized by microsatellite instability (MSI-H), high frequency of expressed neoantigens and good clinical response to immune checkpoint inhibitors. Granzyme B (GrB) is the most abundant serine protease in the granules of cytotoxic T-cells and natural killer cells, mediating anti-tumor immunity. However, recent results confirm a diverse range of physiological functions of GrB including that in extracellular matrix remodelling, inflammation and fibrosis. In the present study, our aim was to investigate whether a frequent genetic variation of *GZMB*, the gene encoding GrB, constituted by three missense single nucleotide polymorphisms (rs2236338, rs11539752 and rs8192917) has any association with cancer risk in individuals with LS. *In silico* analysis and genotype calls from whole exome sequencing data in the Hungarian population confirmed that these SNPs are closely linked. Genotyping results of rs8192917 on a cohort of 145 individuals with LS demonstrated an association of the CC genotype with lower cancer risk. *In silico* prediction proposed likely GrB cleavage sites in a high proportion of shared neontigens in MSI-H tumors. Our results propose the CC genotype of rs8192917 as a potential disease-modifying genetic factor in LS.

## Introduction

Cancer neoantigens are *de novo* amino acid sequences produced by cancer cells provoking antitumor immune response (1). As multiple layers of evidence support the clinically effective modification of the host’s immune system in fighting neoplastic diseases mainly through pharmacologic inhibition of immune checkpoints (2, 3), cutting-edge approaches in oncoimmunology aim to stimulate immune responses by cancer vaccines (4) and *in vitro* modification of effector T cells (5). Although most cancer neoantigens are unique to one’s cancer, several cancers, especially those characterized by microsatellite instability (MSI-H) share several recurrent neoantigens (6, 7), providing a rationale for off-the-shelf cancer vaccines not only in the adjuvant/metastatic setting (8), but also as a part of primary prevention in high-risk individuals (9, 10).

Although MSI-H cancers are sporadic tumors in the majority of the cases, Lynch syndrome (LS), a frequent cancer predisposition syndrome with a prevalence of 1:250-500 significantly elevates the risk of developing MSI-H neoplasms (11). LS is caused by germline pathogenic mutations in genes disrupting the optimal function of the DNA mismatch repair machinery (*MLH1*, *MSH2*, *MSH6*, *PMS2*, *EPCAM*), and is inherited in an autosomal dominant manner. Frequent manifestations include colorectal cancer (CRC) and endometrial cancer, although it mildly elevates the risk of a spectrum of malignancies (12). Specific cancer risks vary by the gene concerned; mutations in *MLH1*, *MSH2* and *EPCAM* result in similarly higher risks for CRC (~50% lifetime risk) and endometrial cancer (~50% lifetime risk) (12). Previous studies have demonstrated high frequencies of effective immunity against the shared neoantigens formerly described in MSI-H tumors not only in LS-associated cancer patients but also in healthy individuals living with LS (13, 14). Optimal immunosurveillance acquires a key part in the host’s prevention of carcinogenesis (15). In healthy patients with LS, normal colonic mucosa exhibits higher frequencies of a wide range of immune cell populations, while this is reduced in LS-associated cancer (16). In colonic premalignant lesions, a higher amount of lymphocyte –activation gene 3 immune checkpoint expression facilitates immune evasion and carcinogenesis (17). Immune-tumor interactions, and immunoediting in particular, are dynamically changing after malignant transformation, the most important of which is the frequent loss of β_2_ microglobulin expression, resulting in impaired HLA class I antigen expression (15, 18–20).

Granzymes are serine proteases, which are the main components of the granules of cytotoxic T-cells and natural killer (NK) cells eliciting perforin-mediated apoptosis of target cells (21). This mechanism is a key effector element in antimicrobial and antitumor immune responses. Granzyme B (GrB) is the most abundant granzyme present in cytotoxic granules, however, recent studies have uncovered several additional molecular mechanisms, in which GrB maintains significant roles (22). In particular, by the expression of GrB in a wide variety of normal epithelial cells and cancer cells, GrB alters extracellular matrix remodeling, epithelial-to-mesenchymal transition and fibrosis (22).

**Figure 1:**
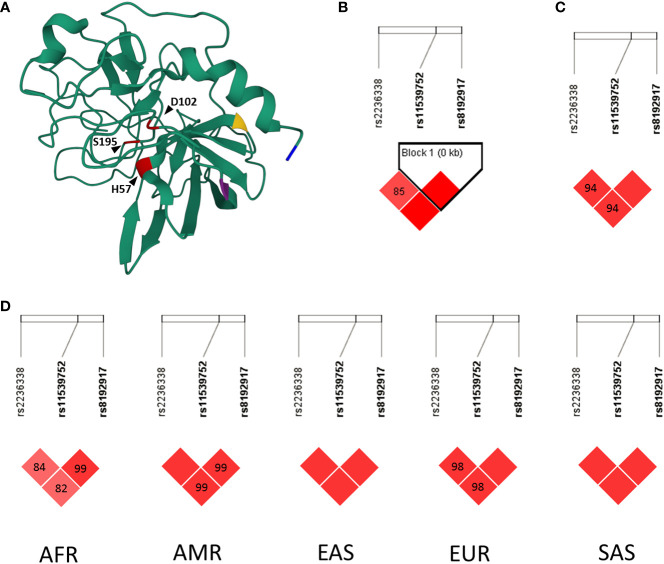
Structural representation and linkage disequilibrium analyses of rs2236338, rs11539752 and rs8192917 of GZMB Panel **(A)**: 3D visualization of the crystallographic structure of Granzyme **(B)** Members of the catalytic triad are highlighted in red (H57, D102, S195). Amino acids altered by rs8192917, rs11539752 and rs2236338 are highlighted in yellow (Q48R), purple (P88A) and blue (Y245H), respectively. 3D protein structure (1FQ3) was deposited to Protein Data Bank (rcsb.org) by Estebanez-Perpina et al. (23) and was visualized using Mol* Viewer (24). Panels **(B–D)**: Linkage disequilibrium (LD) analyses of SNPs rs2236338, rs11539752 and rs8192917. Haploview 4.2 was used for LD analysis of the Hungarian WES cohort (Panel **B**). LDMatrix online tool of the LDLink package was used to analyze LD in the CEU (Utah residents from North and West Europe) population (Panel **C**) and in additional populations (Panel **D**). The D’ values of each SNP pair are indicated within the squares in the matrices. Solid red squares indicate D’ value of 100%. D’ values characterize the extent two which two alleles are nonrandomly associated (25). A D’ value of 100% between two SNPs corresponds to fully dependent inheritance, while a D’ value of 0% signals that the inheritance of the two SNPs are statistically independent from each other. AF, African; AMR, Ad Mixed American; EAS, East Asian; EUR, European; SAS, South Asian.

GrB is a 33 kDa protein, which is encoded by the gene *GZMB* in humans. Former genetic studies have confirmed three closely linked common single nucleotide polymorphisms (SNPs) all resulting in missense mutations (rs8192917 Q48R, rs11539752 P88A and rs2236338 Y245H) (**Figure 1A**) (26, 27). An initial study proposed that Granzyme B harbouring the minor RAH haplotype (resulting from the minor alleles of these SNPs) is incapable of inducing apoptosis (26), however a follow-up study leveraging several layers of evidence including enzyme activity assays dismissed this possibility (27). Nevertheless, there is still no clear evidence on how these three missense alterations, relatively far from the catalytic triad might affect the function of the enzyme.

In the present study, we aimed to analyze the association between rs8192917, a tagging SNP of the RAH haplotype and cancer risk in high-risk individuals with LS. Additionally, based on our *in silico* analysis we anticipate that a significant portion of shared neoantigens in MSI-H cancers can be cleaved by GrB. This study strengthens the immune-related pathogenetic contribution to LS-associated tumorigenesis and invites further investigations in independent LS cohorts to validate the observed disease-modifying nature of rs8192917.

## Materials and methods

### Subjects, DNA isolation and genotyping

145 individuals harboring germline pathogenic mutations of *MLH1*, *MSH2* or terminal deletions of *EPCAM* were consecutively enrolled between 1994 and 2021. Baseline characteristics of study subjects are presented in **Table 1**. Following genetic counseling and written informed consent, DNA was isolated from peripheral blood using the Gentra Puregene Blood Kit (Qiagen, Cat No.: 158389). Mutation analysis was performed using methods described earlier (28, 29) including Sanger sequencing and multiplex ligation-dependent probe amplification (MLPA). The study was approved by the Scientific and Research Ethics Committee of the Medical Research Council of Hungary (ETT-TUKEB 53720-7/2019/EÜIG).

**Table 1:** Baseline characteristics of individuals with LS involved in this study.

n	145
Sex, female (%)	70 (48.3%)
age (years)	42.9 ± 15.2
LS-associated tumor occurrence (%)	110 (75.9%)
age at LS-associated tumor occurrence (years)	39.5 ± 9.4
CRC (% of total)	98 (67.6%)
age at CRC occurrence (years)	40.1 ± 10.9
endometrial cancer (% of women)	28 (40%)
age at endometrial cancer occurrence (years)	47.0 ± 6.5
ovarian cancer (% of women)	1 (1.4%)
age at ovarian cancer occurrence (years)	37.9
cancer of the small intestine (% of total)	3 (2.1%)
age at small intestine cancer occurrence (years)	55.0 ± 8.1
pancreatic cancer (% of total)	2 (1.4%)
age at pancreatic cancer occurrence (years)	45.2 ± 1.9
gastric cancer (% of total)	4 (2.8%)
age at gastric cancer occurrence (years)	50.3 ± 14.5
cancer of the bile ducts (% of total)	1 (0.7%)
age at bile duct cancer occurrence (years)	61.1
cancer of the urinary tract (% of total)	3 (2.1%)
age at urinary cancer occurrence (years)	58.2 ± 8.5
keratoacanthomas of the skin (% of total)	7 (4.8%)
age at skin keratoacanthoma occurrence (years)	45.4 ± 20.0
brain tumor (% of total)	0 (0%)

If multiple cancers in the same organ occurred in the same patient, the age at the first occurrence was included. Data are presented as n (% of total/female subjects) or mean ± standard deviation.

For the genotyping of rs8192917, a predesigned TaqMan Allelic Discrimination assay was used (assay ID: C:_2815152_20). Quantitative real-time PCR was performed on an Applied Biosystems 7500 Fast PCR instrument according to the manufacturer’s instructions.

### Linkage disequilibrium analysis of rs2236338, rs11539752 and rs8192917

To investigate if a tagging SNP from rs2236338, rs11539752 and rs8192917 can be selected we analyzed the linkage disequilibrium of these SNPs. Firstly, LDMatrix online tool (LDLink, version 5.3.3) was used to assess the linkage disequilibrium of these SNPs in various populations (30).

Additionally, to investigate linkage disequilibrium in the same population from which our LS cohort was selected, we leveraged whole exome sequencing (WES) data from our center obtained between 2017-2022. Patients included in this cohort were advised to have their germline DNA subjected to clinical WES based on their individual and family cancer history and the lack of germline pathogenic mutation detection in prior targeted genetic testing. As patients throughout the entire country were included in this database following the recommendations of national centers of excellence, this database can be considered as representative of the current Hungarian population.

WES data of 276 independent individuals were included in this anonymized Hungarian germline sequencing database. WES was performed using standard methods. Sequencing data analysis was performed following the Genome Analysis Toolkit (GATK) best practices guidelines. Paired-end sequencing data were obtained in FASTQ file format and reads were trimmed using Cutadapt to remove adapters and bases where the PHRED quality value was less than 20. The trimmed reads were aligned to Genome Reference Consortium Human Build hg19 using Burrows‐Wheeler Aligner (bwa-mem2-2.0) (31). Picard tools were used to sort, marking duplicates and index reads. Base Quality Score Recalibration (BQSR) was performed using (GATK) (32). Variant discovery was performed in two steps: single-sample variant calling was performed using HaplotypeCaller in GATK; this was followed by GenotypeGVCFs to combine variants from single-sample gVCFs to the multi-sample VCF. Variant annotations were executed using Funcotator. Genotype calls of rs2236338, rs11539752 and rs8192917 were performed as earlier reported (33). Briefly, allele ratios of 0-0.1 and 0.90-1 were considered homozygous, 0.3-0.7 were considered heterozygous, while allele ratios of 0.1-0.3 and 0.7-0.9 were uncalled. The minimum sequencing depth for homozygous calls was 5 reads/allele, and for heterozygous calls was 10 reads/allele. Genotype calls were further analyzed using Haploview 4.2 software (34).

### In silico analysis of Granzyme B cleavage sites in shared frameshift neoantigens

The ability of Granzyme B to cleave shared neoantigens present in MSI-H tumors was analyzed by the PROSPERous online tool (35). Briefly, shared neoantigens which are developed as frameshift peptides due to repeated mismatch repair deficiency provoking an immune response in independent patients with MSI-H tumors were selected based on the prior study of Ruodko et al. (7). The amino acid sequences of the selected neoantigens were subjected to the PROSPERous prediction algorithm using the P4-P2’ cleavage site and logistic regression options.

### Statistical analysis

Statistical analysis was performed using GraphPad Prism 9.0 software. For analyzing the correlation between cancer occurrence and SNP status log-rank test, χ^2^ and Fisher’s exact tests were used. P-values < 0.05 were considered statistically significant. *Post-hoc* power calculations were performed on significant differences observed by Fisher’s exact test to assess the role of sample size. Statistical power > 80% was considered to be strong statistical power.

## Results

### SNPs rs2236338, rs11539752 and rs8192917 are in linkage disequilibrium in the investigated Central European population

We performed a linkage disequilibrium analysis of SNPs rs2236338, rs11539752 and rs8192917. First, we analyzed an anonymized exome sequencing database of Hungarian patients with various medical histories. This cohort includes patients from the whole country and therefore is representative of the current Hungarian population. Within this cohort, the three SNPs were found to be in linkage disequilibrium (**Figure 1B**). Additional *in silico* analysis in the LDmatrix database verified the universal nature of this association in various populations (**Figures 1C, D**).

Since the results indicated an extremely strong linkage disequilibrium between the analyzed three SNPs, we selected rs8192917 as the tagging SNP for this haplotype for further analysis based on the fact that extensive previous research initiatives analyzed its’ association with various conditions and diseases (36–42).

### The minor allele of rs8192917 correlates with delayed cancer occurrence in patients with LS

Our LS cohort included 63 (43.4%), 61 (42.1%) and 21 (14.5%) individuals with germline pathogenic variants in *MLH1*, *MSH2* and *EPCAM* genes, respectively. This distribution correlates well with data from the Prospective Lynch syndrome Database (PLSD), where data from 2607 (51.1%) *MLH1* and 2495 (48.9%) *MSH2* (*EPCAM* carriers were included in the *MSH2* cohort) carriers were analyzed (12).

As expected, the most frequent manifestation of LS was CRC followed by endometrial cancer in women, with more than 75% of the cohort exhibiting any manifestation of LS (**Table 1**). This LS-associated cancer risk corresponds well with data from the PLSD (70-80%), while our cohort contains a larger percentage of CRC cases (67% vs. 50%) possibly due to referral bias (12).

Genotyping of rs8192917 in our LS cohort revealed 85 individuals with TT, 49 individuals with CT and 11 individuals with CC genotype resulting in a minor allele frequency (MAF) of 0.24. This MAF is similar to the MAF of 0.23 observed in the European (non-Finnish) population of the gnomAD database (version 2.1.1) (43).

**Figure 2:**
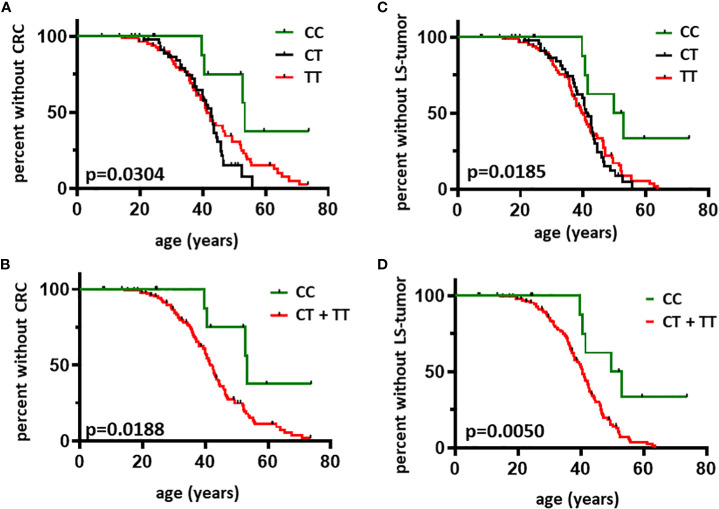
Age-related occurrence of CRC and LS-associated tumors in individuals with LS according to rs8192917 genotype Kaplan-Meier curves were plotted to visualize the first CRC diagnosis based on CC vs. CT vs. TT genotypes (Panel **A**) and CC vs. CT + TT genotypes (Panel **B**). Kaplan-Meier curves were plotted to visualize the first LS-associated tumor diagnosis based on CC vs. CT vs. TT genotypes (Panel **C**) and CC vs. CT + TT genotypes (Panel **D**). LS-associated tumors included malignant tumors of the gastrointestinal tract (CRC, gastric cancer, pancreatic cancer, cancer of the small intestine and of the bile ducts), endometrial cancer, ovarian cancer, malignant tumors of the urinary tract and keratoacanthomas of the skin. Curves were compared by log-rank test. n = 145. P-values < 0.05 were considered statistically significant.

LS individuals harboring the CC genotype were less likely to develop CRC, the main manifestation of the syndrome (**Figures 2A, B**, **Table 2A**). Moreover, individuals homozygous for the minor allele ‘C’ were less likely to develop LS-associated tumor manifestations (**Figures 2C, D**, **Table 2B**). Since LS-associated tumor risks differ in women vs. men mainly due to the risk of endometrial cancer, we performed subgroup analyses based on sex as well (**Supplementary Figure 1**, **Tables 2C, D**) and have found that rs8192917-dependent differences are maintained in the female cohort. Statistical power calculations revealed the associations found in the total cohort to be mild, however statistical power was found to be strong when analyzing LS-associated tumor occurrence restricted to women (**Table 2**). Additional calculations restricted to LS-associated tumor manifestations without CRC or restricted to endometrial cancer occurrence in women have found no associations, probably due to the low sample sizes in these subgroups (**Supplementary Table 1**).

**Table 2:** Analysis of frequencies of CRC and LS-associated tumors in LS individuals based on their rs8192917 genotype.

A total cohort (n = 145)				
genotype	CRC diagnosis	no CRC diagnosis	p-value	*post-hoc* power
CC	4 (36.4%)	7 (63.6%)		
CT	35 (71.4%)	14 (28.6%)		
TT	59 (69.4%)	26 (30.6%)	0.0688	
CC	4 (36.4%)	7 (63.6%)		
CT + TT	94 (70.1%)	40 (29.9%)	**0.0390**	62.8%
B total cohort (n = 145)				
**genotype**	**LS-tumor diagnosis**	**no LS-tumor diagnosis**	**p-value**	***post-hoc* power**
CC	5 (45.5%)	6 (54.5%)		
CT	38 (77.6%)	11 (22.4%)		
TT	67 (78.8%)	18 (21.2%)	**0.0489**	
CC	5 (45.5%)	6 (54.5%)		
CT + TT	105 (78.4%)	29 (21.6%)	**0.0240**	66.6%
C male cohort (n = 75)				
**genotype**	**LS-tumor diagnosis**	**no LS-tumor diagnosis**	**p-value**	***post-hoc* power**
CC	3 (75.0%)	1 (25.0%)		
CT	21 (70.0%)	9 (30.0%)		
TT	30 (73.2%)	11 (26.8%)	0.9487	
CC	3 (75.0%)	1 (25.0%)		
CT + TT	51 (71.8%)	20 (28.2%)	>0.9999	
D female cohort (n = 70)				
**genotype**	**LS-tumor diagnosis**	**no LS-tumor diagnosis**	**p-value**	***post-hoc* power**
CC	2 (28.6%)	5 (71.4%)		
CT	17 (89.5%)	2 (10.5%)		
TT	37 (84.1%)	7 (15.9%)	**0.0014**	
CC	2 (28.6%)	5 (71.4%)		
CT + TT	54 (85.7%)	9 (14.3%)	**0.0027**	92.9%

Panel **(A)** demonstrates individuals with and without CRC diagnosis (n = 145), while Panel **(B–D)** demonstrates individuals with LS-associated tumor diagnosis in the total cohort (Panel **B**, n = 145), in males (Panel **C**, n = 75) and females (Panel **D**, n = 70). LS-associated tumors included malignant tumors of the gastrointestinal tract (CRC, gastric cancer, pancreatic cancer, cancer of the small intestine and of the bile ducts), endometrial cancer, ovarian cancer, malignant tumors of the urinary tract and keratoacanthomas of the skin. 3X2 contingency tables (CC vs. CT vs. TT genotypes) were analyzed by χ^2^ tests, while 2X2 contingency tables (CC vs. CT + TT genotypes) were analyzed by Fisher’s exact tests. P-values < 0.05 were considered statistically significant. In the case of Fisher’s exact tests, *post-hoc* power calculation has additionally been performed. Statistical power > 80% was considered strong.

### Granzyme B is predicted to cleave multiple shared neoantigens in MSI-H cancers

**Figure 3:**
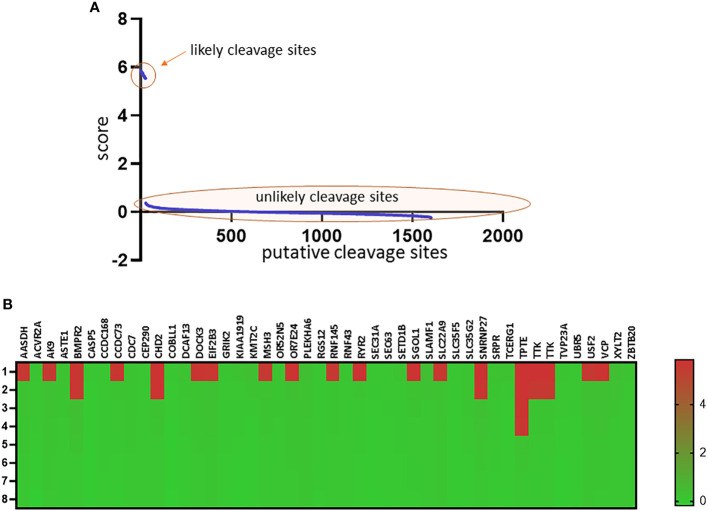
Predicted cleavage sites of Granzyme B in shared neoantigens in MSI-H tumors Representation of each predicted cleavage site score in decreasing order. Sites with the highest probability are located on the left side, sites with the lowest probability are located on the right side (Panel **A**). Heatmap representing the top 8 cleavage sites of each shared neoantigen and their corresponding scores (Panel **B**). On the top of the heatmap gene IDs from which each neoantigen originates are presented. Detailed results of each cleavage site prediction are presented in **Supplementary Table 2**.

Based on the study of Roudko et al, 46 neoantigens shared between MSI-H tumors were included in the *in silico* analysis (7) to assess probable cleavage by Granzyme B (35). Upon the scores of each cleavage sites, putative loci were classified as likely (score>5.0, n = 27) and unlikely (score<1.0, n = 1575) cleavage sites (**Figure 3A**). Applying this threshold, 19 out of 46 (41.3%) shared neoantigens harboured likely cleavage sites (**Figure 3B**, **Supplementary Table 2**).

## Discussion

Identifying disease-modifying genetic factors has tremendous potential in personalizing screening protocols and treatment options, especially in individuals living with high risk for cancer development. Primary studies in individuals living with hereditary predisposition to breast and ovarian cancer found multiple SNPs which may alter cancer risk (44, 45). Based on these results recent studies started to investigate the predictive ability of polygenic risk scores for cancer risk prediction in women living with germline mutations in *BRCA1* and *BRCA2* (46, 47). In LS, earlier studies have found some SNPs which can affect personalized risk of cancer (48–51). In particular, two studies confirmed the association of genomic regions 8q23.3 and 11q23.1 with CRC risk in individuals with LS (48, 51). A recent study found an association between rs2075786, a SNP of the gene encoding telomerase reverse transcriptase (*TERT*) and cancer risk in individuals with germline *MSH2* mutation in a large international cohort (50). Nevertheless, studies leveraging these results to construct polygenic risk scores in this cohort are still scarce. However, a pioneering study in individuals with germline *PMS2* mutations provided a rationale to investigate this matter more thoroughly (49). In anticipation of these studies we aimed to investigate if immunogenetic factors, specifically genetic variants of GrB might associate with cancer risk in LS.

Rs2236338, rs1159752 and rs8192917 are three functionally active SNPs resulting in amino acid change. Jeong et al. previously showed that these three SNPs constitute a haploblock in the Korean population (36). Similarly to this result, by the analysis of the Central European population in the LDMatrix online tool and also in our Hungarian cohort of 276 independent patients, where WES was performed, we found that these three SNPs are closely linked together. Based on these results we selected rs8192917 as a tagging SNP of this haplotype for further genotyping in our LS cohort.

The CC genotype of rs8192917 was associated with decreased risk for LS-associated tumors in our cohort. This association was significant when the analysis was restricted to females, while in males a tendency toward this association was verified. The association of the CC genotype with decreased cancer occurrence was also validated when the analysis was restricted to CRC, but not to endometrial cancer and other, less frequent LS manifestations, possibly due to the insufficient sample size in this regard. Earlier, the ‘C’ allele of rs8192917 has been associated with vitiligo, an autoimmune dermatologic condition in three independent cohorts (36–38). Moreover, rs8192917 has also been associated with subacute sclerosing panencephalitis (39) and postoperative keloids (40) in single cohorts. Additionally, this SNP has been associated with transplantation outcomes after HLA-matched unrelated bone marrow transplantation (41). On the functional level, a study has suggested that rs8192917 associates with natural killer cell cytotoxicity (42), however further functional validation is needed to determine the specific nature of this finding. Nonetheless, another *GZMB* SNP, independent of rs8192917 has been associated with joint destruction in rheumatoid arthritis (52), strengthening the role of genetic variants of *GZMB* in immune-related pathogenic mechanisms.

Although these previous lines of evidence suggest a wide-raging disease-modifying effect of GZMB genetic variants, mechanistic insights are still lacking. Sun et al. have clearly demonstrated that the minor RAH haplotype retains the pro-apoptotic activity of GrB (27), however, GrB has multiple roles in extracellular matrix remodeling, epithelial-to-mesenchymal transition, inflammation and fibrosis (22). QPY and RAH variants may have different substrate-specificities, which might directly affect some of these mechanisms. Regarding autoimmune pathomechanisms as in the case of generalized vitiligo, it has already been suggested that altered cleavage of autoantigens might explain the previously confirmed association of rs8192917 with this disease (38). Transposing this hypothesis to MSI-H carcinogenesis in LS, our *in silico* analysis revealed that a large proportion of shared neoantigens in MSI-H cancers encompass likely GrB cleavage sites. Differential cleavage of these frameshift peptides serving as neoantigens might directly affect optimal immunosurveillance, a key feature in LS-related tumorigenesis (15). Moreover, performing in-depth analyses regarding autoimmune conditions and cancer risk in individuals with LS might further shed light on the immunological pathomechanism of these diseases which can possibly be affected by GrB and other mediators.

It is important to note the limitations of our study. Deriving from its’ monocentric nature, we cannot infer the observed association to other populations. Although our cohort is smaller than those of large international consortia, such as the PLSD (12), it is still the largest Hungarian Lynch syndrome cohort ever studied and is comparable to a Swedish national study, where the country’s population is also comparable to Hungary (53). By disregarding individuals with *MSH6* and *PMS2* mutations, where cancer risk is significantly lower, and selecting only individuals with *MLH1*, *MSH2* or *EPCAM* mutations, where lifetime colorectal cancer risk is approximately 50%, we were able to perform our comparative study in a relatively homogeneous population.

In conclusion, we found that rs8192917 SNP of the gene encoding GrB correlates with cancer risk in our LS cohort. Following validation of this finding in independent cohorts, this SNP can be included in personalized risk stratification and screening recommendations in affected individuals. Further research avenues might also include the functional assessment of the QPY and RAH variants of GrB to investigate possible differences in substrate-specificities that might explain the observed protective effect.

## Data availability statement

The datasets presented in this study can be found in online repositories. The names of the repository/repositories and accession number(s) can be found below: European Variation Archive, Project ID: PRJEB55654, Analysis ID: ERZ13336597.

## Ethics statement

The studies involving human participants were reviewed and approved by Scientific and Research Ethics Committee of the Medical Research Council of Hungary. Written informed consent to participate in this study was provided by the participants’ legal guardian/next of kin.

## Author contributions

Hypothesis and objective: VG and AP. Genetic counseling: VG, HB, AP, and EO. Molecular genetic diagnosis: AB, TP, JP, HB, AP, and EO. Genotyping: PN and VG. Bioinformatics analysis of WES: IL. In silico analysis: VG. Manuscript preparation: VG, IL, AB, JP, HB, EO, and AP. All authors contributed to the article and approved the submitted version.
