# Supplementary material for: A common genetic variation in GZMB may associate with cancer risk in patients with Lynch syndrome
Source: Front Oncol. 2023 Feb 20;13:1005066. doi: 10.3389/fonc.2023.1005066 (PMC9986427; doi:10.3389/fonc.2023.1005066)
Supplement: Supplementary file 1 [file DataSheet_1.pdf]

frontiers

Supplementary Material

Supplementary Figure 1 – Age-related occurrence of LS-associated tumors in women and men with LS according to rs8192917 genotype Kaplan-Meier curves were plotted to visualize the first LS-associated tumor diagnosis based on CC vs. CT vs. TT genotypes (Panels A and C) and CC vs. CT + TT genotypes (Panels B and D). LS-associated tumors included malignant tumors of the gastrointestinal tract (CRC, gastric cancer, pancreatic cancer, cancer of the small intestine and of the bile ducts), endometrial cancer, ovarian cancer, malignant tumors of the urinary tract and keratoacanthomas of the skin. Panels A and B are restricted to male subjects (n = 75), while panels C and D are restricted to female subjects (n = 70). Curves were compared by log-rank test. P-values < 0.05 were considered statistically significant.

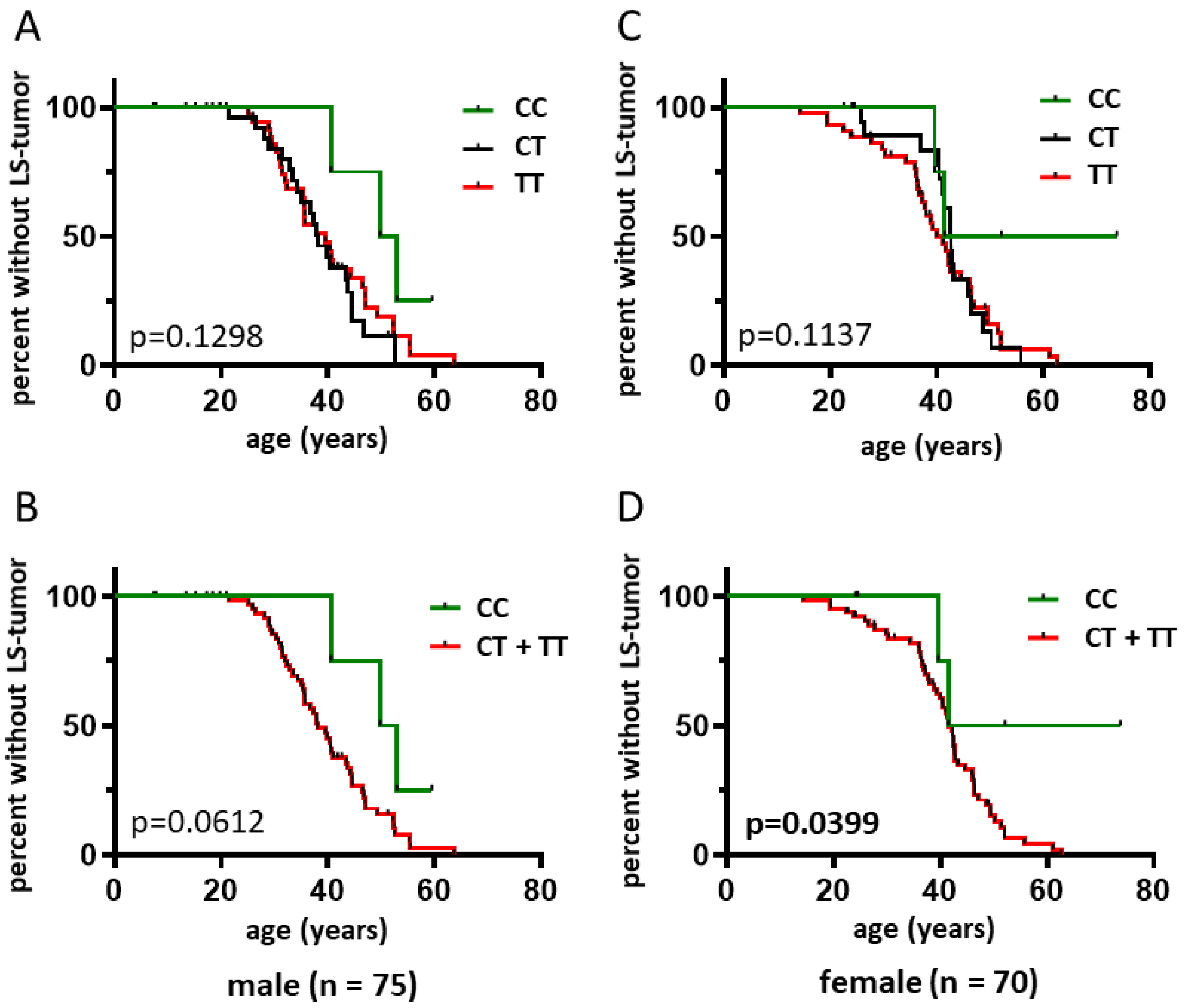

Supplementary Table 1 - Analysis of frequencies of LS-associated tumors without CRC and of endometrial cancer in LS individuals based on their rs8192917 genotype Panel A demonstrates individuals with and without LS-associated tumor diagnosis without the inclusion of CRC diagnosis (n = 145). In this case, LS-associated tumors included malignant tumors of the gastrointestinal tract (gastric cancer, pancreatic cancer, cancer of the small intestine and of the bile ducts), endometrial cancer, ovarian cancer, malignant tumors of the urinary tract and keratoacanthomas of the skin. Panel B demonstrates women with or without endometrial cancer diagnosis (n = 70). 3X2 contingency tables (CC vs. CT vs. TT genotypes) were analyzed by χ^2 tests, while 2X2 contingency tables (CC vs. CT + TT genotypes) were analyzed by Fisher's exact tests. P-values < 0.05 were considered statistically significant.

A

total cohort (n = 145)

genotype	LS-tumor (without CRC) diagnosis	no LS-tumor (without CRC) diagnosis	p-value
CC	2	9	
CT	11	38	
TT	28	57	0.3190

CC	2	9	
CT + TT	39	95	0.7286

B

female cohort (n = 70)

genotype	endometrial cancer diagnosis	no endometrial cancer diagnosis	p-value
CC	1	6	
CT	10	9	
TT	17	27	0.1993

CC	1	6	
CT + TT	27	36	0.2298

Supplementary Table 2 - Detailed results of each cleavage site prediction. Extended data for Figure 4.

gene ID	neoantigen protein sequence	rank (within same neontigen)	position	site	score
AASDH	KDGEIFFWDEKTVRSNVMANVLTLNLCNRLLKSFSKWSLVQLHGIIRKN	1	9	FFWDEK	5.591
		2	20	VMANVL	0.241
		3	24	VLTLNL	0.231
		4	43	VQLHGI	0.167
		5	16	VRSNVM	0.147
		6	34	LKSFSK	0.123
		7	37	FSKWSL	0.089
		8	8	IFFWDE	0.072
		9	42	LVQLHG	0.054
		10	22	ANVLTL	0.044
		11	5	DGEIFF	0.032
		12	40	WSLVQL	0.031
		13	29	LCNRLL	0.024
		14	6	GEIFFW	0.021
		15	14	KTVRSN	0.019
		16	45	LHGIIR	0.018
		17	11	WDEKTV	0.016
		18	33	LLKSFS	0.014
		19	32	RLLKSF	0.014
		20	12	DEKTVR	0.004
		21	4	KDGEIF	-0.003
		22	25	LTLNLC	-0.013
		23	27	LNLCNR	-0.028
		24	18	SNVMAN	-0.035
		25	7	EIFFWD	-0.046
		26	31	NRLLKS	-0.052
		27	36	SFSKWS	-0.053
		28	41	SLVQLH	-0.055
		29	13	EKTVRS	-0.061
		30	39	KWSLVQ	-0.067
		31	17	RSNVMA	-0.099
		32	28	NLCNRL	-0.101
		33	21	MANVLT	-0.106
		34	23	NVLTLN	-0.107
		35	46	HGIIRK	-0.119

		36	35	KSFSKW	-0.120
		37	38	SKWSLV	-0.142
		38	44	QLHGII	-0.147
		39	26	TLNLCN	-0.150
		40	19	NVMANV	-0.157
		41	47	GIIRKN	-0.162
		42	30	CNRLLK	-0.178
		43	15	TVRSNV	-0.182
		44	10	FWDEKT	-0.182
ACVR2A	MQEVVVHKKRGLF	1	7	VVVHKK	0.163
		2	9	VHKKRG	0.111
		3	5	QEVVVH	0.090
		4	8	VVHKKR	0.075
		5	4	MQEVVV	-0.002
		6	6	EVVVHK	-0.058
		7	10	HKKRGL	-0.069
		8	11	KKRGLF	-0.173
AK9	RREVSSFFSKQGLTLLPRAGYSGTIIAHCNLELLGSRDPPTSASQSARITGMSHHTQPLPSGLRHSC NSFSRLTLL	1	39	GSRDPP	5.558
		2	35	LELLGS	0.255
		3	7	VSSFFF	0.229
		4	53	ITGMSH	0.203
		5	19	LLPRAG	0.198
		6	5	REVSSF	0.159
		7	22	RAGYSG	0.153
		8	62	PLPSGL	0.127
		9	37	LLGSRD	0.105
		10	46	SASQSA	0.102
		11	44	PTSASQ	0.092
		12	4	RREVSS	0.089
		13	38	LGSRDP	0.085
		14	30	IAHCNL	0.081
		15	42	DPPTSA	0.072
		16	34	NLELLG	0.053
		17	36	ELLGSR	0.049
		18	41	RDPPTS	0.041
		19	15	QGLTLL	0.041
		20	14	KQGLTL	0.040
		21	64	PSGLRH	0.035
		22	29	IIAHCN	0.032
		23	61	QPLPSG	0.032
		24	65	SGLRHS	0.017
		25	26	SGTIIA	0.014

		26	21	PRAGYS	0.008
		27	45	TSASQS	0.002
		28	60	TQPLPS	0.000
		29	43	PPTSAS	-0.001
		30	17	LTLLPR	-0.003
		31	75	SRLTLL	-0.005
		32	47	ASQSAR	-0.006
		33	8	SSFFFS	-0.010
		34	71	CNSFSR	-0.012
		35	25	YSGTII	-0.014
		36	9	SFFFSK	-0.017
		37	12	FSKQGL	-0.017
		38	63	LPSGLR	-0.018
		39	69	HSCNSF	-0.027
		40	74	FSRLTL	-0.029
		41	23	AGYSGT	-0.031
		42	50	SARITG	-0.035
		43	66	GLRHSC	-0.040
		44	32	HCNLEL	-0.043
		45	6	EVSSFF	-0.044
		46	20	LPRAGY	-0.045
		47	72	NSFSRL	-0.048
		48	11	FFSKQG	-0.052
		49	33	CNLELL	-0.053
		50	49	QSARIT	-0.056
ASTE1	RSNSKKKGRRNRIPAVLRTEGEPLHTPSVGMRETTGLGC	1	32	VGMRET	0.217
		2	19	VLRTEG	0.207
		3	22	TEGEPL	0.160
		4	35	RETTGL	0.121
		5	24	GEPLHT	0.106
		6	27	LHTPSV	0.075
		7	20	LRTEGE	0.058
		8	16	IPAVLR	0.046
		9	15	RIPAVL	0.037
		10	28	HTPSVG	0.030
		11	23	EGEPLH	0.028
		12	34	MRETTG	0.024
		13	31	SVGMRE	0.021
		14	18	AVLRTE	0.011
		15	37	TTGLGC	0.006
		16	17	PAVLRT	-0.005
		17	36	ETTGLG	-0.006
		18	30	PSVGMR	-0.009

		19	21	RTEGEP	-0.035
		20	6	NSKKKG	-0.049
		21	26	PLHTPS	-0.062
		22	29	TPSVGM	-0.089
		23	9	KKGRRN	-0.089
		24	5	SNSKKK	-0.094
		25	25	EPLHTP	-0.112
		26	14	NRIPAV	-0.122
		27	10	KGRRNR	-0.127
		28	7	SKKKGR	-0.137
		29	4	RSNSKK	-0.153
		30	13	RNRIPA	-0.171
		31	33	GMRETT	-0.189
		32	11	GRRNRI	-0.196
		33	12	RRNRIP	-0.209
		34	8	KKKGRR	-0.213
BMPR2	STPLTIGEKTEIQLTMNDSKHKLESPALKQVSPASPPTQQPQTPQDSRQVLA	1	46	TPQDSR	5.663
		2	18	TMNDSK	5.636
		3	34	VSPASP	0.345
		4	26	LESPAL	0.299
		5	9	IGEKTE	0.220
		6	31	LKQVSP	0.082
		7	6	PLTIGE	0.062
		8	10	GEKTEI	0.061
		9	27	ESPALK	0.038
		10	37	ASPPTQ	0.034
		11	12	KTEIQL	0.031
		12	4	STPLTI	0.011
		13	17	LTMNDS	0.007
		14	15	IQLTMN	0.002
		15	33	QVSPAS	-0.001
		16	45	QTPQDS	-0.001
		17	7	LTIGEK	-0.002
		18	13	TEIQLT	-0.007
		19	44	PQTPQD	-0.016
		20	29	PALKQV	-0.019
		21	25	KLESPA	-0.029
		22	36	PASPPT	-0.030
		23	49	DSRQVL	-0.030
		24	30	ALKQVS	-0.032
		25	48	QDSRQV	-0.036
		26	24	HKLESP	-0.038

		27	21	DSKHKL	-0.038
		28	16	QLTMND	-0.048
		29	50	SRQVLA	-0.048
		30	20	NDSKHK	-0.059
		31	8	TIGEKT	-0.065
		32	38	SPPTQQ	-0.072
		33	28	SPALKQ	-0.073
		34	23	KHKLES	-0.077
		35	5	TPLTIG	-0.078
		36	42	QQPQTP	-0.081
		37	32	KQVSPA	-0.081
		38	40	PTQQPQ	-0.085
		39	14	EIQLTM	-0.086
		40	22	SKHKLE	-0.094
		41	39	PPTQQP	-0.117
		42	47	PQDSRQ	-0.122
		43	11	EKTEIQ	-0.122
		44	35	SPASPP	-0.130
		45	41	TQQPQT	-0.139
		46	43	QPQTPQ	-0.176
		47	19	MNDSKH	-0.180
CASP5	KDNHKKKQLRCWNTWAKMFFMVFLIIWQNTMF	1	25	VFLIIW	0.128
		2	29	IWQNTM	-0.023
		3	28	IIWQNT	-0.050
		4	27	LIIWQN	-0.054
		5	22	FFMVFL	-0.060
		6	12	LRCWNT	-0.063
		7	23	FMVFLI	-0.073
		8	15	WNTWAK	-0.077
		9	21	MFFMVF	-0.097
		10	17	TWAKMF	-0.097
		11	18	WAKMFF	-0.097
		12	24	MVFLII	-0.099
		13	7	HKKKQL	-0.114
		14	19	AKMFFM	-0.114
		15	4	KDNHKK	-0.121
		16	10	KQLRCW	-0.133
		17	26	FLIIWQ	-0.151
		18	9	KKQLRC	-0.154
		19	20	KMFFMV	-0.157
		20	16	NTWAKM	-0.165
		21	11	QLRCWN	-0.168
		22	14	CWNTWA	-0.169

		23	30	WQNTMF	-0.176
		24	5	DNHKKK	-0.188
		25	8	KKKQLR	-0.192
		26	6	NHKKKQ	-0.195
		27	13	RCWNTW	-0.204
CCDC168	KQNRPFFLPVYRQTHWRLYPKPFAGLFPLKP	1	23	PKPFAG	0.121
		2	27	AGLFPL	0.076
		3	29	LFPLKP	0.053
		4	6	NRPFFL	0.041
		5	13	VYRQTH	0.029
		6	21	LYPKPF	0.025
		7	26	FAGLFP	-0.008
		8	11	LPVYRQ	-0.015
		9	25	PFAGLF	-0.017
		10	10	FLPVYR	-0.036
		11	24	KPFAGL	-0.060
		12	9	FFLPVY	-0.086
		13	16	QTHWRL	-0.088
		14	8	PFFLPV	-0.094
		15	12	PVYRQT	-0.106
		16	28	GLFPLK	-0.111
		17	19	WRLYPK	-0.122
		18	22	YPKPFA	-0.148
		19	17	THWRLY	-0.150
		20	20	RLYPKP	-0.162
		21	14	YRQTHW	-0.171
		22	15	RQTHWR	-0.174
		23	5	QNRPFF	-0.180
		24	7	RPFFLP	-0.181
		25	4	KQNRPF	-0.186
		26	18	HWRLYP	-0.194
CCDC73	SSLDIKKILFHVRNIVYGIQVMLC	1	4	SSLDIK	5.663
		2	19	VYGIQV	0.185
		3	15	VRNIVY	0.124
		4	22	IQVMLC	0.063
		5	11	ILFHVR	0.024
		6	6	LDIKKI	0.012
		7	8	IKKILF	0.004
		8	18	IVYGIQ	-0.027
		9	21	GIQVML	-0.039
		10	17	NIVYGI	-0.047
		11	7	DIKKIL	-0.088

		12	12	LFHVRN	-0.089
		13	20	YGIQVM	-0.093
		14	5	SLDIKK	-0.105
		15	10	KILFHV	-0.114
		16	13	FHVRNI	-0.119
		17	16	RNIVYG	-0.121
		18	9	KKILFH	-0.166
		19	14	HVRNIV	-0.194
CDC7	RFQAEGSLKKTSRILNLQVLKKILRSFMKLYHSLVMCLRLRTKLEKALSALFIWPQHSYK	1	22	VLKKIL	0.183
		2	47	LEKALS	0.167
		3	6	QAEGSL	0.154
		4	38	VMCLRL	0.145
		5	7	AEGSLK	0.140
		6	48	EKALSA	0.112
		7	51	LSALFI	0.110
		8	56	IWPQHS	0.102
		9	46	KLEKAL	0.082
		10	18	LNLQVL	0.080
		11	20	LQVLKK	0.043
		12	32	KLYHSL	0.043
		13	49	KALSAL	0.041
		14	43	LRTKLE	0.040
		15	36	SLVMCL	0.036
		16	11	LKKTSR	0.033
		17	8	EGSLKK	0.023
		18	17	ILNLQV	0.013
		19	25	KILRSF	0.007
		20	27	LRSFMK	0.003
		21	41	LRLRTK	-0.004
		22	50	ALSALF	-0.008
		23	13	KTSRIL	-0.011
		24	5	FQAEGS	-0.019
		25	26	ILRSFM	-0.021
		26	28	RSFMKL	-0.025
		27	57	WPQHSY	-0.028
		28	4	RFQAEG	-0.034
		29	37	LVMCLR	-0.037
		30	31	MKLYHS	-0.056
		31	23	LKKILR	-0.057
		32	10	SLKKTS	-0.057
		33	52	SALFIW	-0.058
		34	15	SRILNL	-0.064
		35	9	GSLKKT	-0.066

		36	54	LFIWPQ	-0.067
		37	33	LYHSLV	-0.069
		38	45	TKLEKA	-0.077
		39	35	HSLVMC	-0.082
		40	19	NLQVLK	-0.085
		41	53	ALFIWP	-0.088
		42	42	RLRTKL	-0.093
		43	39	MCLRLR	-0.095
		44	34	YHSLVM	-0.100
		45	21	QVLKKI	-0.103
		46	44	RTKLEK	-0.109
		47	14	TSRILN	-0.116
		48	29	SFMKLY	-0.123
		49	58	PQHSYK	-0.131
		50	24	KKILRS	-0.132
CEP290	EQVKHFFFHESSLFKLPGFLLLLVTISIFILYVIFEK	1	19	LPGFLL	0.128
		2	26	LVTISI	0.122
		3	29	ISIFIL	0.112
		4	6	VKHFFF	0.098
		5	11	FHESSL	0.093
		6	34	LYVIFE	0.087
		7	27	VTISIF	0.087
		8	20	PGFLLL	0.085
		9	25	LLVTIS	0.079
		10	12	HESSLF	0.059
		11	23	LLLLVT	0.059
		12	14	SSLFKL	0.051
		13	18	KLPGFL	0.050
		14	24	LLLVTI	0.046
		15	33	ILYVIF	0.016
		16	13	ESSLFK	0.009
		17	16	LFKLPG	0.008
		18	21	GFLLLL	0.007
		19	31	IFILYV	-0.012
		20	10	FFHESS	-0.027
		21	28	TISIFI	-0.048
		22	22	FLLLLV	-0.052
		23	9	FFFHES	-0.065
		24	32	FILYVI	-0.068
		25	4	EQVKHF	-0.069
		26	17	FKLPGF	-0.086
		27	30	SIFILY	-0.095

		28	15	SLFKLP	-0.103
		29	8	HFFFHE	-0.111
		30	35	YVIFEK	-0.113
		31	5	QVKHFF	-0.145
		32	7	KHFFFH	-0.164
CHD2	STFFLFVFFLGEKPQLTIVYLDRHGLLSVLLCFSNLDSFFKA	1	22	VYLDRH	5.793
		2	37	SNLDSF	5.739
		3	32	VLLCFS	0.229
		4	10	VFFLGE	0.198
		5	29	LLSVLL	0.137
		6	8	LFVFFL	0.113
		7	24	LDRHGL	0.105
		8	13	LGEKPQ	0.103
		9	39	LDSFFK	0.092
		10	27	HGLLSV	0.091
		11	30	LSVLLC	0.086
		12	33	LLCFSN	0.077
		13	14	GEKPQL	0.076
		14	19	LTIVYL	0.040
		15	28	GLLSVL	0.019
		16	34	LCFSNL	0.015
		17	21	IVYLDR	0.004
		18	20	TIVYLD	-0.001
		19	26	RHGLLS	-0.022
		20	15	EKPQLT	-0.035
		21	40	DSFFKA	-0.043
		22	36	FSNLDS	-0.046
		23	18	QLTIVY	-0.052
		24	17	PQLTIV	-0.053
		25	12	FLGEKP	-0.055
		26	9	FVFFLG	-0.057
		27	35	CFSNLD	-0.063
		28	11	FFLGEK	-0.071
		29	31	SVLLCF	-0.071
		30	6	FFLFVF	-0.072
		31	23	YLDRHG	-0.086
		32	25	DRHGLL	-0.087
		33	4	STFFLF	-0.090
		34	7	FLFVFF	-0.097
		35	5	TFFLFV	-0.113
		36	38	NLDSFF	-0.120
		37	16	KPQLTI	-0.126
COBLL1	AK PS SF FC [illegible]	1	4	AKPSSF	0.089

		2	6	PSSFFC	0.016
		3	15	REYRVT	0.004
		4	14	RREYRV	-0.088
		5	7	SSFFCR	-0.102
		6	5	KPSSFF	-0.119
		7	12	RCRREY	-0.128
		8	11	CRCRRE	-0.143
		9	8	SFFCRC	-0.150
		10	16	EYRVTM	-0.157
		11	9	FFCRCR	-0.198
		12	10	FCRCRR	-0.201
		13	13	CRREYR	-0.215
DCAF13	FNPIEVMFFLSMFYLLWLNNFSSV	1	7	IEVMFF	0.248
		2	9	VMFFLS	0.149
		3	21	LNNFSS	0.097
		4	13	LSMFYL	0.084
		5	6	PIEVMF	0.008
		6	4	FNPIEV	0.005
		7	8	EVMFFL	-0.004
		8	10	MFFLSM	-0.024
		9	18	LLWLNN	-0.035
		10	16	FYLLWL	-0.036
		11	19	LWLNNF	-0.038
		12	12	FLSMFY	-0.039
		13	14	SMFYLL	-0.040
		14	22	NNFSSV	-0.052
		15	17	YLLWLN	-0.073
		16	20	WLNNFS	-0.112
		17	15	MFYLLW	-0.128
		18	11	FFLSMF	-0.129
		19	5	NPIEVM	-0.186
DOCK3	FHHPLGDTPQPSLPGPCASLLSTLSQPPPQAPSQVWTAATLRCPAVPAAACPP	1	7	PLGDTP	5.728
		2	17	PGPCAS	0.187
		3	49	VPAAAC	0.185
		4	24	LSTLSQ	0.159
		5	38	VWTAAT	0.158
		6	21	ASLLST	0.109
		7	47	PAVPAA	0.097
		8	48	AVPAAA	0.095
		9	23	LLSTLS	0.093
		10	32	PQAPSQ	0.087
		11	19	PCASLL	0.051

		12	16	LPGPCA	0.050
		13	11	TPQPSL	0.044
		14	5	HHPLGD	0.021
		15	39	WTAATL	0.020
		16	22	SLLSTL	0.019
		17	18	GPCASL	0.016
		18	30	PPPQAP	0.013
		19	14	PSLPGP	0.012
		20	8	LGDTPQ	0.007
		21	20	CASLLS	0.001
		22	27	LSQPPP	-0.003
		23	36	SQVWTA	-0.005
		24	44	LRCPAV	-0.008
		25	12	PQPSLP	-0.009
		26	50	PAAACP	-0.011
		27	10	DTPQPS	-0.017
		28	51	AAACPP	-0.027
		29	35	PSQVWT	-0.033
		30	33	QAPSQV	-0.035
		31	15	SLPGPC	-0.044
		32	40	TAATLR	-0.050
		33	29	QPPPQA	-0.051
		34	41	AATLRC	-0.055
		35	45	RCPAVP	-0.057
		36	28	SQPPPQ	-0.057
		37	31	PPQAPS	-0.058
		38	13	QPSLPG	-0.060
		39	34	APSQVW	-0.070
		40	9	GDTPQP	-0.072
		41	37	QVWTAA	-0.074
		42	42	ATLRCP	-0.081
		43	46	CPAVPA	-0.086
		44	43	TLRCPA	-0.105
		45	26	TLSQPP	-0.109
		46	25	STLSQP	-0.110
		47	4	FHHPLG	-0.118
		48	6	HPLGDT	-0.145
EIF2B3	PGQKGKKKQWSSVT SLEWTAQERGCSSW LMKQTWMKSWSLR DPSYRSILEYVSTRVL WMPTSTV	1	42	SLRDPS	5.616
		2	55	VSTRVL	0.282
		3	16	VTSLEW	0.231
		4	52	LEYVST	0.221
		5	51	ILEYVS	0.191
		6	14	SSVTSL	0.174

		7	25	ERGCSS	0.152
		8	19	LEWTAQ	0.148
		9	38	MKSWSL	0.123
		10	59	VLWMPT	0.099
		11	46	PSYRSI	0.069
		12	43	LRDPSY	0.068
		13	18	SLEWTA	0.051
		14	10	KKQWSS	0.046
		15	61	WMPTST	0.033
		16	4	PGQKGK	0.031
		17	13	WSSVTS	0.017
		18	24	QERGCS	0.006
		19	23	AQERGC	0.002
		20	60	LWMPTS	-0.009
		21	26	RGCSSW	-0.019
		22	40	SWSLRD	-0.024
		23	48	YRSILE	-0.026
		24	27	GCSSWL	-0.032
		25	44	RDPSYR	-0.032
		26	15	SVTSLE	-0.035
		27	20	EWTAQE	-0.043
		28	22	TAQERG	-0.044
		29	21	WTAQER	-0.049
		30	28	CSSWLM	-0.055
		31	49	RSILEY	-0.058
		32	11	KQWSSV	-0.059
		33	36	TWMKSW	-0.061
		34	41	WSLRDP	-0.061
		35	6	QKGKKK	-0.074
		36	17	TSLEWT	-0.082
		37	32	LMKQTW	-0.083
		38	45	DPSYRS	-0.084
		39	53	EYVSTR	-0.087
		40	29	SSWLMK	-0.089
		41	30	SWLMKQ	-0.089
		42	57	TRVLWM	-0.094
		43	35	QTWMKS	-0.095
		44	50	SILEYV	-0.100
		45	7	KGKKKQ	-0.102
		46	47	SYRSIL	-0.102
		47	12	QWSSVT	-0.103
		48	54	YVSTRV	-0.117

		49	58	RVLWMP	-0.119
		50	62	MPTSTV	-0.123
GRIK2	GEFLYKSKKTLNWKREPRLSYLKTMYSSLFWSQFPFLQCCHHHHLHHHYHHVLNKYI	1	18	REPRLS	0.147
		2	55	VLNKYI	0.108
		3	4	GEFLYK	0.032
		4	25	LKTMYS	0.032
		5	31	SLFWSQ	0.027
		6	26	KTMYSS	0.021
		7	22	LSYLKT	0.010
		8	27	TMYSSL	0.010
		9	20	PRLSYL	0.008
		10	30	SSLFWS	0.003
		11	17	KREPRL	-0.018
		12	19	EPRLSY	-0.020
		13	5	EFLYKS	-0.020
		14	14	LNWKRE	-0.030
		15	35	SQFPFL	-0.031
		16	29	YSSLFW	-0.033
		17	9	KSKKTL	-0.039
		18	6	FLYKSK	-0.039
		19	36	QFPFLQ	-0.049
		20	21	RLSYLK	-0.070
		21	32	LFWSQF	-0.071
		22	34	WSQFPF	-0.073
		23	28	MYSSLF	-0.079
		24	7	LYKSKK	-0.082
		25	38	PFLQCC	-0.087
		26	40	LQCCHH	-0.089
		27	51	HYHHVL	-0.092
		28	48	LHHHYH	-0.113
		29	53	HHVLNK	-0.114
		30	23	SYLKTM	-0.114
		31	54	HVLNKY	-0.117
		32	8	YKSKKT	-0.121
		33	33	FWSQFP	-0.122
		34	39	FLQCCH	-0.128
		35	15	NWKREP	-0.130
		36	13	TLNWKR	-0.135
		37	12	KTLNWK	-0.142
		38	11	KKTLNW	-0.143
		39	24	YLKTMY	-0.143
		40	43	CHHHHL	-0.148
		41	10	SKKTLN	-0.158

		42	47	HLHHHY	-0.162
		43	37	FPFLQC	-0.170
		44	46	HHLHHH	-0.187
		45	52	YHHVLN	-0.194
		46	44	HHHHLH	-0.214
		47	50	HHYHHV	-0.216
		48	41	QCCHHH	-0.218
		49	16	WKREPR	-0.220
		50	42	CCHHHH	-0.228
KIAA1919	YMFLVSVIFFVCF	1	8	VSVIFF	0.238
		2	10	VIFFVC	0.127
		3	7	LVSVIF	0.029
		4	5	MFLVSV	0.029
		5	11	IFFVCF	-0.011
		6	6	FLVSVI	-0.039
		7	4	YMFLVS	-0.080
		8	9	SVIFFV	-0.111
KMT2C	VSVEPKKRNKKTKLWFSLINIHHRKNPLLPMR	1	6	VEPKKR	0.337
		2	4	VSVEPK	0.180
		3	16	KLWFSL	0.058
		4	17	LWFSLI	-0.038
		5	15	TKLWFS	-0.039
		6	5	SVEPKK	-0.041
		7	30	PLLPMR	-0.050
		8	28	KNPLLP	-0.050
		9	24	IHHRKN	-0.056
		10	19	FSLINI	-0.060
		11	22	INIHHR	-0.067
		12	21	LINIHH	-0.092
		13	12	NKKTKL	-0.092
		14	18	WFSLIN	-0.107
		15	27	RKNPLL	-0.109
		16	8	PKKRNK	-0.126
		17	26	HRKNPL	-0.137
		18	13	KKTKLW	-0.143
		19	14	KTKLWF	-0.147
		20	29	NPLLPM	-0.152
		21	20	SLINIH	-0.160
		22	10	KRNKKT	-0.189
		23	7	EPKKRN	-0.190
		24	11	RNKKTK	-0.200
		25	23	NIHHRK	-0.202

		26	9	KKRNKK	-0.209
		27	25	HHRKNP	-0.236
MSH3	NKENVRDKKRATFLLALWECSLPQARLCLIVSRTLLLVQS	1	7	NVRDKK	5.537
		2	20	LWECSL	0.236
		3	34	VSRTLL	0.204
		4	33	IVSRTL	0.140
		5	17	LLALWE	0.105
		6	18	LALWEC	0.091
		7	38	LLLVQS	0.080
		8	30	LCLIVS	0.079
		9	8	VRDKKR	0.072
		10	5	KENVRD	0.034
		11	15	TFLLAL	0.033
		12	25	LPQARL	0.020
		13	24	SLPQAR	0.017
		14	35	SRTLLL	-0.001
		15	12	KRATFL	-0.005
		16	13	RATFLL	-0.007
		17	21	WECSLP	-0.017
		18	32	LIVSRT	-0.019
		19	14	ATFLLA	-0.021
		20	26	PQARLC	-0.023
		21	37	TLLLVQ	-0.036
		22	28	ARLCLI	-0.042
		23	23	CSLPQA	-0.043
		24	31	CLIVSR	-0.043
		25	6	ENVRDK	-0.056
		26	27	QARLCL	-0.063
		27	22	ECSLPQ	-0.066
		28	19	ALWECS	-0.068
		29	4	NKENVR	-0.077
		30	16	FLLALW	-0.084
		31	36	RTLLLV	-0.086
		32	9	RDKKRA	-0.101
		33	10	DKKRAT	-0.123
		34	29	RLCLIV	-0.134
		35	11	KKRATF	-0.173
OR52N5	HHPMYFFLA MLSPSLTSLP APPLYPMHSA SSGSVSKKLTS MLAWPRCSL	1	16	PSLTSL	0.181
		2	58	VWSLGC	0.175
		3	56	SQVWSL	0.140
		4	13	MLSPSL	0.137

		5	37	VSKKLT	0.132
		6	30	HSASSG	0.123
		7	34	SGSVSK	0.122
		8	32	ASSGSV	0.113
		9	60	SLGCSC	0.105
		10	11	LAMLSP	0.103
		11	14	LSPSLT	0.093
		12	26	LYPMHS	0.086
		13	41	LTSMLA	0.084
		14	33	SSGSVS	0.075
		15	29	MHSASS	0.066
		16	18	LTSLPA	0.063
		17	22	PAPPLY	0.062
		18	21	LPAPPL	0.059
		19	52	LFMGSQ	0.048
		20	61	LGCSCS	0.040
		21	62	GCSCSW	0.034
		22	31	SASSGS	0.029
		23	36	SVSKKL	0.018
		24	46	AWPRCS	0.017
		25	10	FLAMLS	0.008
		26	47	WPRCSL	0.007
		27	54	MGSQVW	0.001
		28	50	CSLFMG	-0.010
		29	51	SLFMGS	-0.012
		30	6	PMYFFL	-0.015
		31	19	TSLPAP	-0.026
		32	39	KKLTSM	-0.028
		33	23	APPLYP	-0.028
		34	55	GSQVWS	-0.029
		35	15	SPSLTS	-0.030
		36	20	SLPAPP	-0.034
		37	59	WSLGCS	-0.034
		38	42	TSMLAW	-0.035
		39	27	YPMHSA	-0.036
		40	35	GSVSKK	-0.038
		41	28	PMHSAS	-0.039
		42	9	FFLAML	-0.049
		43	40	KLTSML	-0.049
		44	45	LAWPRC	-0.051
		45	12	AMLSPS	-0.057
		46	44	MLAWPR	-0.057

		47	4	HHPMYF	-0.074
		48	63	CSCSWL	-0.074
		49	38	SKKLTS	-0.079
		50	53	FMGSQV	-0.083
OR7E24	MSYFPILFFFSSKGVRATQSHRISQVSQNSSSWDSQRIQNCSRSSLGCSCPCTWSRCWGTCSSSWLSALTPTSTPPCTSSSPTCPWLTSVSPPPRSPR	1	34	SSWDSQ	5.730
		2	29	VSQNSS	0.315
		3	93	VSPPPR	0.214
		4	18	VRATQS	0.196
		5	26	ISQVSQ	0.186
		6	72	LTPTST	0.165
		7	90	LTSVSP	0.133
		8	61	WGTCSS	0.120
		9	10	LFFFSS	0.118
		10	48	SLGCSC	0.105
		11	95	PPPRSP	0.089
		12	66	SSWLSA	0.087
		13	79	PCTSSS	0.087
		14	41	IQNCSR	0.086
		15	9	ILFFFS	0.083
		16	69	LSALTP	0.082
		17	43	NCSRSS	0.069
		18	54	PCTWSR	0.055
		19	33	SSSWDS	0.052
		20	88	PWLTSV	0.049
		21	65	SSSWLS	0.044
		22	44	CSRSSL	0.041
		23	78	PPCTSS	0.027
		24	16	KGVRAT	0.025
		25	67	SWLSAL	0.018
		26	63	TCSSSW	0.001
		27	62	GTCSSS	0.001
		28	71	ALTPTS	-0.001
		29	30	SQNSSS	-0.003
		30	64	CSSSWL	-0.004
		31	15	SKGVRA	-0.008
		32	32	NSSSWD	-0.011
		33	77	TPPCTS	-0.011
		34	80	CTSSSP	-0.012
		35	47	SSLGCS	-0.012
		36	45	SRSSLG	-0.013
		37	8	PILFFF	-0.016
		38	83	SSPTCP	-0.019
		39	13	FSSKGV	-0.019

		40	31	QNSSSW	-0.022
		41	19	RATQSH	-0.023
		42	49	LGCSCP	-0.024
		43	86	TCPWLT	-0.025
		44	52	SCPCTW	-0.026
		45	46	RSSLGC	-0.029
		46	94	SPPPRS	-0.035
		47	6	YFPILF	-0.036
		48	85	PTCPWL	-0.037
		49	89	WLTSVS	-0.041
		50	82	SSSPTC	-0.041
PLEKHA6	WVNLRRGYPRLKTFGVPLGSILCLAGSLSTMAPTPPSTPMIISTTRQECGRRASVPCRPMIGSARPGPWRTSAMPSAMGV ALPTSCESGRSPPATGGRMPPSGSQAPPGSQSIMMSWMPPLAPCAACPCSPAPTLCPAHPARAPTAVPAFTPLSAHPVP VLSGCHLAVRTSMLTLLPM	1	161	VPVLSG	0.337
		2	19	VPLGSI	0.233
		3	83	VALPTS	0.225
		4	124	LAPCAA	0.196
		5	171	VRTSML	0.181
		6	164	LSGCHL	0.178
		7	5	VNLRRG	0.172
		8	111	PGSQSI	0.149
		9	28	AGSLST	0.146
		10	150	VPAFTP	0.142
		11	24	ILCLAG	0.142
		12	163	VLSGCH	0.141
		13	90	ESGRSP	0.131
		14	153	FTPLSA	0.129
		15	27	LAGSLS	0.129
		16	26	CLAGSL	0.119
		17	169	LAVRTS	0.117
		18	36	PTPPST	0.117
		19	80	AMGVAL	0.114
		20	84	ALPTSC	0.110
		21	129	ACPCSP	0.101
		22	21	LGSILC	0.096
		23	20	PLGSIL	0.095
		24	64	IGSARP	0.092
		25	101	RMPPSG	0.087
		26	86	PTSCES	0.087
		27	104	PSGSQA	0.082
		28	160	PVPVLS	0.081
		29	31	LSTMAP	0.076
		30	25	LCLAGS	0.073
		31	78	PSAMGV	0.072

		32	109	APPGSQ	0.071
		33	89	CESGRS	0.071
		34	69	PGPWRT	0.066
		35	18	GVPLGS	0.060
		36	87	TSCESG	0.056
		37	156	LSAHPV	0.051
		38	103	PPSGSQ	0.050
		39	108	QAPPGS	0.050
		40	75	SAMPSA	0.049
		41	17	FGVPLG	0.049
		42	95	PPATGG	0.044
		43	45	ISTTRQ	0.043
		44	134	PAPTLC	0.039
		45	62	PMIGSA	0.032
		46	58	VPCRPM	0.032
		47	138	LCPAHP	0.032
		48	44	IISTTR	0.032
		49	105	SGSQAP	0.028
		50	71	PWRTSA	0.026
RGS12	ESIAKIGKKNIRKLIWTKQRSFLSLFPKLRATEQMTNVGC	1	23	RSFLSL	0.091
		2	32	LRATEQ	0.058
		3	6	IAKIGK	0.054
		4	9	IGKKNI	0.042
		5	35	TEQMTN	0.040
		6	28	LFPKLR	0.039
		7	26	LSLFPK	0.039
		8	20	TKQRSF	-0.007
		9	5	SIAKIG	-0.010
		10	30	PKLRAT	-0.011
		11	18	IWTKQR	-0.013
		12	27	SLFPKL	-0.016
		13	14	IRKLIW	-0.020
		14	22	QRSFLS	-0.032
		15	34	ATEQMT	-0.038
		16	17	LIWTKQ	-0.056
		17	25	FLSLFP	-0.057
		18	4	ESIAKI	-0.069
		19	37	QMTNVG	-0.071
		20	8	KIGKKN	-0.075
		21	31	KLRATE	-0.085
		22	24	SFLSLF	-0.092
		23	21	KQRSFL	-0.095
		24	12	KNIRKL	-0.097

		25	38	MTNVGC	-0.108
		26	16	KLIWTK	-0.122
		27	7	AKIGKK	-0.130
		28	36	EQMTNV	-0.132
		29	19	WTKQRS	-0.137
		30	15	RKLIWT	-0.141
		31	33	RATEQM	-0.141
		32	29	FPKLRA	-0.153
		33	13	NIRKLI	-0.161
		34	10	GKKNIR	-0.195
		35	11	KKNIRK	-0.196
RNF145	VFSKKKKKKQHGCKGETGGSVKCGPEGAKHHAVGCPVQMGCQLLFPADPKK	1	50	FPADPK	5.571
		2	38	VGCPVQ	0.171
		3	30	PEGAKH	0.146
		4	4	VFSKKK	0.146
		5	20	GETGGS	0.120
		6	26	VKCGPE	0.112
		7	21	ETGGSV	0.104
		8	19	KGETGG	0.102
		9	48	LLFPAD	0.089
		10	28	CGPEGA	0.072
		11	42	VQMGCQ	0.069
		12	49	LFPADP	0.046
		13	27	KCGPEG	0.044
		14	22	TGGSVK	0.034
		15	24	GSVKCG	0.018
		16	43	QMGCQL	0.010
		17	44	MGCQLL	0.004
		18	37	AVGCPV	-0.005
		19	15	QHGCKG	-0.007
		20	18	CKGETG	-0.016
		21	23	GGSVKC	-0.017
		22	16	HGCKGE	-0.021
		23	31	EGAKHH	-0.021
		24	29	GPEGAK	-0.030
		25	41	PVQMGC	-0.033
		26	47	QLLFPA	-0.049
		27	39	GCPVQM	-0.062
		28	51	PADPKK	-0.067
		29	33	AKHHAV	-0.071
		30	40	CPVQMG	-0.083
		31	45	GCQLLF	-0.086

		32	35	HHAVGC	-0.088
		33	36	HAVGCP	-0.096
		34	32	GAKHHA	-0.101
		35	34	KHHAVG	-0.102
		36	46	CQLLFP	-0.108
		37	25	SVKCGP	-0.109
		38	5	FSKKKK	-0.119
		39	17	GCKGET	-0.121
		40	13	KKQHGC	-0.128
		41	12	KKKQHG	-0.140
		42	6	SKKKKK	-0.157
		43	10	KKKKKQ	-0.182
		44	7	KKKKKK	-0.183
		44	8	KKKKKK	-0.183
		44	9	KKKKKK	-0.183
		45	14	KQHGCK	-0.200
		46	11	KKKKQH	-0.205
RNF43	PQRKRRGVPPSPPLALGPRMQLCTQLARFFPITPPVWHILGPQRHTP	1	39	VWHILG	0.150
		2	11	VPPSPP	0.136
		3	19	LGPRMQ	0.116
		4	35	ITPPVW	0.116
		5	13	PSPPLA	0.106
		6	14	SPPLAL	0.094
		7	42	ILGPQR	0.088
		8	43	LGPQRH	0.080
		9	10	GVPPSP	0.061
		10	16	PLALGP	0.041
		11	25	LCTQLA	0.025
		12	15	PPLALG	0.000
		13	17	LALGPR	-0.002
		14	12	PPSPPL	-0.003
		15	9	RGVPPS	-0.010
		16	29	LARFFP	-0.023
		17	38	PVWHIL	-0.027
		18	18	ALGPRM	-0.029
		19	28	QLARFF	-0.039
		20	37	PPVWHI	-0.048
		21	32	FFPITP	-0.062
		22	26	CTQLAR	-0.074
		23	24	QLCTQL	-0.075
		24	34	PITPPV	-0.081
		25	23	MQLCTQ	-0.084
		26	36	TPPVWH	-0.088

		27	8	RRGVPP	-0.096
		28	20	GPRMQL	-0.103
		29	30	ARFFPI	-0.110
		30	27	TQLARF	-0.115
		31	21	PRMQLC	-0.115
		32	45	PQRHTP	-0.118
		33	5	QRKRRG	-0.124
		34	41	HILGPQ	-0.147
		35	4	PQRKRR	-0.149
		36	40	WHILGP	-0.154
		37	22	RMQLCT	-0.164
		38	6	RKRRGV	-0.165
		39	44	GPQRHT	-0.168
		40	7	KRRGVP	-0.175
		41	31	RFFPIT	-0.193
		42	33	FPITPP	-0.225
RYR2	VLGHYNNFFLPLTFSTLLWDSRH	1	20	LLWDSR	5.781
		2	4	VLGHYN	0.193
		3	14	PLTFST	0.080
		4	15	LTFSTL	0.053
		5	13	LPLTFS	0.026
		6	19	TLLWDS	-0.000
		7	12	FLPLTF	-0.002
		8	5	LGHYNN	-0.004
		9	16	TFSTLL	-0.010
		10	18	STLLWD	-0.018
		11	17	FSTLLW	-0.056
		12	10	NFFLPL	-0.073
		13	8	YNNFFL	-0.092
		14	21	LWDSRH	-0.109
		15	11	FFLPLT	-0.117
		16	9	NNFFLP	-0.149
		17	7	HYNNFF	-0.191
		18	6	GHYNNF	-0.203
SEC31A	INYCQKKLMLLRLNLRKMCG PF	1	11	LMLLRL	0.066
		2	13	LLRLNL	0.044
		3	18	LRKMCG	0.002
		4	9	KKLMLL	-0.021
		5	16	LNLRKM	-0.024
		6	14	LRLNLR	-0.029
		7	4	INYCQK	-0.033
		8	12	MLLRLN	-0.060

		9	6	YCQKKL	-0.064
		10	8	QKKLML	-0.093
		11	10	KLMLLR	-0.117
		12	19	RKMCGP	-0.154
		13	15	RLNLRK	-0.173
		14	17	NLRKMC	-0.176
		15	7	CQKKLM	-0.191
		16	5	NYCQKK	-0.196
		17	20	KMCGPF	-0.202
SEC63	KKKPLKKNLHLCYYHSQSNRNKSRQMESLGMKLQ	1	29	MESLGM	0.111
		2	31	SLGMKL	0.080
		3	32	LGMKLQ	0.033
		4	27	RQMESL	0.014
		5	12	LHLCYY	-0.005
		6	26	SRQMES	-0.008
		7	14	LCYYHS	-0.014
		8	28	QMESLG	-0.019
		9	7	PLKKNL	-0.021
		10	17	YHSQSN	-0.033
		11	30	ESLGMK	-0.047
		12	5	KKPLKK	-0.055
		13	22	NRNKSR	-0.078
		14	8	LKKNLH	-0.086
		15	15	CYYHSQ	-0.087
		16	25	KSRQME	-0.094
		17	9	KKNLHL	-0.108
		18	18	HSQSNR	-0.124
		19	24	NKSRQM	-0.124
		20	19	SQSNRN	-0.128
		21	11	NLHLCY	-0.128
		22	10	KNLHLC	-0.130
		23	21	SNRNKS	-0.135
		24	20	QSNRNK	-0.140
		25	6	KPLKKN	-0.151
		26	16	YYHSQS	-0.156
		27	13	HLCYYH	-0.164
		28	4	KKKPLK	-0.176
		29	23	RNKSRQ	-0.229
SETD1B	MENSHPPTTTTSSPRRSPALRARGGTTIGEVTS	1	31	IGEVTS	0.221
		2	23	LRARGG	0.117
		3	11	TTTTSS	0.053
		4	15	SSPRRS	0.045

		5	21	PALRAR	0.025
		6	27	GGTTIG	0.011
		7	18	RRSPAL	0.004
		8	26	RGGTTI	0.003
		9	10	PTTTTS	-0.004
		10	28	GTTIGE	-0.004
		11	12	TTTSSP	-0.019
		12	19	RSPALR	-0.023
		13	25	ARGGTT	-0.025
		14	4	MENSHP	-0.033
		15	30	TIGEVT	-0.038
		16	20	SPALRA	-0.049
		17	22	ALRARG	-0.055
		18	7	SHPPTT	-0.064
		19	16	SPRRSP	-0.074
		20	14	TSSPRR	-0.076
		21	9	PPTTTT	-0.092
		22	8	HPPTTT	-0.095
		23	29	TTIGEV	-0.101
		24	17	PRRSPA	-0.104
		25	5	ENSHPP	-0.109
		26	13	TTSSPR	-0.119
		27	24	RARGGT	-0.140
		28	6	NSHPPT	-0.150
SGOL1	KTVPQKKCTNLSVPMMLTILIWKRVFILLLSDKK	1	32	LLSDKK	5.751
		2	30	ILLLSD	0.250
		3	28	VFILLL	0.188
		4	16	VPMMLT	0.089
		5	6	VPQKKC	0.081
		6	15	SVPMML	0.057
		7	14	LSVPMM	0.034
		8	31	LLLSDK	0.015
		9	24	IWKRVF	0.010
		10	22	ILIWKR	0.009
		11	26	KRVFIL	-0.010
		12	18	MMLTIL	-0.034
		13	29	FILLLS	-0.036
		14	11	CTNLSV	-0.038
		15	20	LTILIW	-0.044
		16	19	MLTILI	-0.049
		17	5	TVPQKK	-0.051
		18	27	RVFILL	-0.054

		19	23	LIWKRV	-0.073
		20	17	PMMLTI	-0.081
		21	10	KCTNLS	-0.091
		22	13	NLSVPM	-0.098
		23	4	KTVPQK	-0.098
		24	21	TILIWK	-0.113
		25	9	KKCTNL	-0.114
		26	12	TNLSVP	-0.116
		27	7	PQKKCT	-0.127
		28	25	WKRVFI	-0.156
		29	8	QKKCTN	-0.177
SLAMF1	QTTVEKKALRSMPKSRNQVLFRRNLTPSQLRTLAPPYMLLPQSLSQSLSRKQIPSQSMLV	1	7	VEKKAL	0.372
		2	42	LLPQSL	0.258
		3	28	LTPSQL	0.137
		4	46	SLSQSL	0.132
		5	56	IPSQSM	0.111
		6	44	PQSLSQ	0.099
		7	22	VLFRRN	0.097
		8	47	LSQSLS	0.073
		9	36	LAPPYM	0.064
		10	37	APPYML	0.051
		11	14	SMPKSR	0.046
		12	48	SQSLSR	0.044
		13	57	PSQSML	0.037
		14	33	LRTLAP	0.031
		15	10	KALRSM	0.019
		16	27	NLTPSQ	0.003
		17	43	LPQSLS	0.000
		18	6	TVEKKA	-0.001
		19	31	SQLRTL	-0.001
		20	12	LRSMPK	-0.007
		21	23	LFRRNL	-0.007
		22	41	MLLPQS	-0.017
		23	51	LSRKQI	-0.017
		24	55	QIPSQS	-0.028
		25	38	PPYMLL	-0.030
		26	45	QSLSQS	-0.031
		27	30	PSQLRT	-0.031
		28	50	SLSRKQ	-0.044
		29	58	SQSMLV	-0.046
		30	9	KKALRS	-0.047
		31	16	PKSRNQ	-0.056
		32	18	SRNQVL	-0.057

		33	4	QTTVEK	-0.064
		34	35	TLAPPY	-0.065
		35	54	KQIPSQ	-0.066
		36	20	NQVLFR	-0.072
		37	5	TTVEKK	-0.077
		38	13	RSMPKS	-0.081
		39	39	PYMLLP	-0.099
		40	49	QSLSRK	-0.101
		41	32	QLRTLA	-0.102
		42	53	RKQIPS	-0.110
		43	26	RNLTPS	-0.118
		44	19	RNQVLF	-0.126
		45	21	QVLFRR	-0.128
		46	29	TPSQLR	-0.131
		47	40	YMLLPQ	-0.134
		48	8	EKKALR	-0.137
		49	11	ALRSMP	-0.141
		50	34	RTLAPP	-0.145
SLC22A9	KKELEAAQKKNLLCVKCSTCPTYVKGSPSCPLRDLQTLWPILALISMSSIWGTMFSCCRLSLVQSSSWPTVLHLGH	1	34	PLRDLQ	5.632
		2	66	VQSSSW	0.244
		3	27	VKGSPS	0.209
		4	47	LISMSS	0.189
		5	7	LEAAQK	0.172
		6	65	LVQSSS	0.143
		7	48	ISMSSI	0.143
		8	44	ILALIS	0.138
		9	45	LALISM	0.137
		10	5	KELEAA	0.130
		11	74	VLHLGH	0.129
		12	64	SLVQSS	0.113
		13	41	LWPILA	0.107
		14	63	LSLVQS	0.104
		15	53	IWGTMF	0.076
		16	28	KGSPSC	0.069
		17	70	SWPTVL	0.059
		18	18	VKCSTC	0.055
		19	72	PTVLHL	0.054
		20	6	ELEAAQ	0.050
		21	16	LCVKCS	0.044
		22	4	KKELEA	0.035
		23	50	MSSIWG	0.032
		24	32	SCPLRD	0.020

		25	60	CCRLSL	0.017
		26	54	WGTMFS	0.012
		27	36	RDLQTL	0.010
		28	24	PTYVKG	-0.018
		29	25	TYVKGS	-0.018
		30	38	LQTLWP	-0.025
		31	30	SPSCPL	-0.025
		32	55	GTMFSC	-0.027
		33	73	TVLHLG	-0.029
		34	15	LLCVKC	-0.031
		35	8	EAAQKK	-0.032
		36	43	PILALI	-0.032
		37	42	WPILAL	-0.037
		38	68	SSSWPT	-0.037
		39	29	GSPSCP	-0.037
		40	62	RLSLVQ	-0.045
		41	35	LRDLQT	-0.049
		42	14	NLLCVK	-0.052
		43	58	FSCCRL	-0.053
		44	33	CPLRDL	-0.053
		45	22	TCPTYV	-0.053
		46	51	SSIWGT	-0.053
		47	17	CVKCST	-0.054
		48	9	AAQKKN	-0.056
		49	40	TLWPIL	-0.058
		50	10	AQKKNL	-0.061
SLC35F5	AKISFFFALCGFWQICHIKKHFQTHKLL	1	6	ISFFFA	0.101
		2	12	LCGFWQ	0.044
		3	7	SFFFAL	0.003
		4	18	ICHIKK	-0.020
		5	21	IKKHFQ	-0.024
		6	9	FFALCG	-0.028
		7	10	FALCGF	-0.032
		8	25	FQTHKL	-0.050
		9	13	CGFWQI	-0.060
		10	5	KISFFF	-0.068
		11	26	QTHKLL	-0.077
		12	11	ALCGFW	-0.098
		13	4	AKISFF	-0.120
		14	8	FFFALC	-0.157
		15	16	WQICHI	-0.161
		16	15	FWQICH	-0.164
		17	24	HFQTHK	-0.171

		18	20	HIKKHF	-0.183
		19	22	KKHFQT	-0.183
		20	17	QICHIK	-0.186
		21	14	GFWQIC	-0.190
		22	23	KHFQTH	-0.198
		23	19	CHIKKH	-0.222
SLC35G2	KGLLSEMKKKGELSLEPWIPYLHQQKTQ	1	18	LEPWIP	0.215
		2	13	KGELSL	0.211
		3	4	KGLLSE	0.146
		4	14	GELSLE	0.120
		5	7	LSEMKK	0.107
		6	15	ELSLEP	0.017
		7	16	LSLEPW	0.017
		8	8	SEMKKK	0.016
		9	6	LLSEMK	0.011
		10	12	KKGELS	-0.009
		11	17	SLEPWI	-0.025
		12	20	PWIPYL	-0.046
		13	22	IPYLHQ	-0.048
		14	11	KKKGEL	-0.050
		15	21	WIPYLH	-0.051
		16	5	GLLSEM	-0.051
		17	25	LHQQKT	-0.061
		18	10	MKKKGE	-0.062
		19	9	EMKKKG	-0.077
		20	23	PYLHQQ	-0.081
		21	26	HQQKTQ	-0.139
		22	19	EPWIPY	-0.142
		23	24	YLHQQK	-0.170
SNRNP27	RIEVLKDDFFPLILVREWILYFVFNLHSKNRISVLLSCKVRKSYL	1	7	VLKDDF	5.837
		2	8	LKDDFF	5.666
		3	5	IEVLKD	0.293
		4	18	VREWIL	0.279
		5	35	ISVLLS	0.179
		6	43	VRKSYL	0.147
		7	37	VLLSCK	0.132
		8	16	ILVREW	0.131
		9	26	VFNLHS	0.115
		10	38	LLSCKV	0.058
		11	22	ILYFVF	0.048
		12	15	LILVRE	0.047
		13	36	SVLLSC	0.038

		14	12	FFPLIL	0.027
		15	19	REWILY	0.016
		16	6	EVLKDD	0.001
		17	39	LSCKVR	-0.002
		18	34	RISVLL	-0.009
		19	10	DDFFPL	-0.012
		20	27	FNLHSK	-0.014
		21	24	YFVFNL	-0.025
		22	29	LHSKNR	-0.041
		23	42	KVRKSY	-0.047
		24	23	LYFVFN	-0.048
		25	14	PLILVR	-0.050
		26	41	CKVRKS	-0.057
		27	4	RIEVLK	-0.064
		28	32	KNRISV	-0.066
		29	17	LVREWI	-0.070
		30	33	NRISVL	-0.073
		31	21	WILYFV	-0.078
		32	9	KDDFFP	-0.088
		33	13	FPLILV	-0.113
		34	31	SKNRIS	-0.114
		35	20	EWILYF	-0.119
		36	11	DFFPLI	-0.135
		37	30	HSKNRI	-0.158
		38	40	SCKVRK	-0.161
		39	28	NLHSKN	-0.163
		40	25	FVFNLH	-0.168
SRPR	KAKNSKKRGPRRKVLMVLWLPANQSLQKSQVFQWVLRTE	1	34	VFQWVL	0.235
		2	20	VLWLPA	0.156
		3	17	VLMVLW	0.128
		4	24	PANQSL	0.106
		5	18	LMVLWL	0.090
		6	28	SLQKSQ	0.028
		7	21	LWLPAN	0.021
		8	23	LPANQS	0.021
		9	16	KVLMVL	0.012
		10	13	PRRKVL	-0.018
		11	11	RGPRRK	-0.021
		12	27	QSLQKS	-0.029
		13	37	WVLRTE	-0.034
		14	22	WLPANQ	-0.040
		15	4	KAKNSK	-0.041
		16	31	KSQVFQ	-0.066

		17	19	MVLWLP	-0.070
		18	7	NSKKRG	-0.070
		19	29	LQKSQV	-0.071
		20	32	SQVFQW	-0.072
		21	26	NQSLQK	-0.086
		22	30	QKSQVF	-0.087
		23	25	ANQSLQ	-0.098
		24	10	KRGPRR	-0.098
		25	15	RKVLMV	-0.104
		26	36	QWVLRT	-0.109
		27	8	SKKRGP	-0.130
		28	6	KNSKKR	-0.133
		29	5	AKNSKK	-0.150
		30	35	FQWVLR	-0.158
		31	33	QVFQWV	-0.161
		32	12	GPRRKV	-0.177
		33	14	RRKVLM	-0.183
		34	9	KKRGPR	-0.221
TCERG1	EHIEALTKKRESTLGNFWMKLLQLP	1	6	IEALTK	0.236
		2	13	RESTLG	0.125
		3	12	KRESTL	0.000
		4	14	ESTLGN	-0.000
		5	22	MKLLQL	-0.005
		6	17	LGNFWM	-0.009
		7	9	LTKKRE	-0.010
		8	4	EHIEAL	-0.025
		9	16	TLGNFW	-0.035
		10	7	EALTKK	-0.048
		11	10	TKKRES	-0.063
		12	5	HIEALT	-0.064
		13	8	ALTKKR	-0.069
		14	20	FWMKLL	-0.079
		15	19	NFWMKL	-0.080
		16	11	KKREST	-0.084
		17	15	STLGNF	-0.090
		18	23	KLLQLP	-0.097
		19	21	WMKLLQ	-0.148
		20	18	GNFWMK	-0.152
TPTE	LLVDVVYIFLT LSCLGIFPDGH IYFDFYDLLFC	1	20	IFPDGH	5.802
		2	4	LLVDVV	5.791
		3	26	IYFDFY	5.709
		4	29	DFYDLL	5.636

		5	6	VDVVYI	0.237
		6	8	VVYIFL	0.198
		7	9	VYIFLT	0.087
		8	11	IFLTLS	0.086
		9	22	PDGHIY	0.069
		10	18	LGIFPD	0.065
		11	13	LTLSCL	0.060
		12	15	LSCLGI	0.045
		13	14	TLSCLG	0.020
		14	28	FDFYDL	0.015
		15	12	FLTLSC	0.015
		16	5	LVDVVY	0.009
		17	17	CLGIFP	-0.034
		18	31	YDLLFC	-0.037
		19	10	YIFLTL	-0.053
		20	7	DVVYIF	-0.067
		21	19	GIFPDG	-0.071
		22	23	DGHIYF	-0.088
		23	16	SCLGIF	-0.095
		24	27	YFDFYD	-0.098
		25	24	GHIYFD	-0.099
		26	30	FYDLLF	-0.124
		27	25	HIYFDF	-0.150
		28	21	FPDGHI	-0.198
TTK	KTFEKKRGKNDLQLFVMSDTTYKIYWTVILLNPCGNLHLKTTSL	1	19	VMSDTT	5.845
		2	11	GKNDLQ	5.548
		3	17	LFVMSD	0.195
		4	42	LKTTSL	0.159
		5	31	VILLNP	0.129
		6	34	LNPCGN	0.071
		7	6	FEKKRG	0.040
		8	29	WTVILL	0.025
		9	15	LQLFVM	0.013
		10	33	LLNPCG	0.011
		11	32	ILLNPC	0.007
		12	27	IYWTVI	-0.007
		13	9	KRGKND	-0.018
		14	37	CGNLHL	-0.025
		15	36	PCGNLH	-0.029
		16	40	LHLKTT	-0.032
		17	13	NDLQLF	-0.039
		18	10	RGKNDL	-0.039
		19	21	SDTTYK	-0.053

		20	28	YWTVIL	-0.054
		21	16	QLFVMS	-0.066
		22	14	DLQLFV	-0.067
		23	20	MSDTTY	-0.074
		24	5	TFEKKR	-0.079
		25	12	KNDLQL	-0.084
		26	41	HLKTTS	-0.094
		27	22	DTTYKI	-0.100
		28	7	EKKRGK	-0.104
		29	38	GNLHLK	-0.109
		30	35	NPCGNL	-0.123
		31	39	NLHLKT	-0.132
		32	26	KIYWTV	-0.134
		33	30	TVILLN	-0.139
		34	23	TTYKIY	-0.144
		35	4	KTFEKK	-0.154
		36	18	FVMSDT	-0.156
		37	24	TYKIYW	-0.166
		38	25	YKIYWT	-0.186
		39	8	KKRGKN	-0.200
TTK	SSSSKTFEKKGEKNDLQLFVMSDTTYKIYWTVILLNPCGNLHLKTTSL	1	23	VMSDTT	5.845
		2	15	EKNDLQ	5.571
		3	21	LFVMSD	0.195
		4	46	LKTTSL	0.159
		5	35	VILLNP	0.129
		6	10	FEKKGE	0.079
		7	14	GEKNDL	0.077
		8	38	LNPCGN	0.071
		9	13	KGEKND	0.051
		10	33	WTVILL	0.025
		11	19	LQLFVM	0.013
		12	37	LLNPCG	0.011
		13	36	ILLNPC	0.007
		14	9	TFEKKG	0.004
		15	31	IYWTVI	-0.007
		16	5	SSSKTF	-0.020
		17	41	CGNLHL	-0.025
		18	6	SSKTFE	-0.028
		19	40	PCGNLH	-0.029
		20	44	LHLKTT	-0.032
		21	17	NDLQLF	-0.039
		22	4	SSSSKT	-0.049

		23	25	SDTTYK	-0.053
		24	32	YWTVIL	-0.054
		25	7	SKTFEK	-0.060
		26	20	QLFVMS	-0.066
		27	18	DLQLFV	-0.067
		28	24	MSDTTY	-0.074
		29	12	KKGEKN	-0.079
		30	16	KNDLQL	-0.084
		31	45	HLKTTS	-0.094
		32	11	EKKGEK	-0.095
		33	26	DTTYKI	-0.100
		34	42	GNLHLK	-0.109
		35	39	NPCGNL	-0.123
		36	43	NLHLKT	-0.132
		37	30	KIYWTV	-0.134
		38	34	TVILLN	-0.139
		39	27	TTYKIY	-0.144
		40	8	KTFEKK	-0.154
		41	22	FVMSDT	-0.156
		42	28	TYKIYW	-0.166
		43	29	YKIYWT	-0.186
TVP23A	MIWIVFFLAPYFP	1	8	VFFLAP	0.144
		2	11	LAPYFP	0.096
		3	7	IVFFLA	0.065
		4	6	WIVFFL	0.016
		5	5	IWIVFF	-0.006
		6	10	FLAPYF	-0.062
		7	4	MIWIVF	-0.102
		8	9	FFLAPY	-0.119
UBR5	NMQNRQKKKGKNSPCCQKKLRVQNQGHLLMILLH N	1	31	LLMILL	0.077
		2	25	VQNQGH	0.075
		3	27	NQGHLL	0.035
		4	12	KGKNSP	-0.009
		5	30	HLLMIL	-0.010
		6	23	LRVQNQ	-0.010
		7	15	NSPCCQ	-0.015
		8	11	KKGKNS	-0.024
		9	32	LMILLH	-0.055
		10	18	CCQKKL	-0.067
		11	26	QNQGHL	-0.089
		12	24	RVQNQG	-0.089
		13	28	QGHLLM	-0.097
		14	33	MILLHN	-0.097

		15	21	KKLRVQ	-0.100
		16	29	GHLLMI	-0.104
		17	8	RQKKKG	-0.114
		18	17	PCCQKK	-0.124
		19	14	KNSPCC	-0.136
		20	7	NRQKKK	-0.142
		21	9	QKKKGK	-0.156
		22	5	MQNRQK	-0.157
		23	22	KLRVQN	-0.161
		24	16	SPCCQK	-0.172
		25	20	QKKLRV	-0.176
		26	4	NMQNRQ	-0.183
		27	19	CQKKLR	-0.186
		28	10	KKKGKN	-0.189
		29	13	GKNSPC	-0.205
		30	6	QNRQKK	-0.209
USF2	QLCDNTCPLFFPPLVEKLMEPEHPEMRGEEPSTTKWSGGGGTRSTTGSSSFRKSFQTVTQTTARRERV KEGSCPRPAITSGSCARPTSACRRPSKRPSGCRWTTSS	1	4	QLCDNT	5.544
		2	18	VEKLME	0.310
		3	71	VKEGSC	0.277
		4	31	GEEPST	0.210
		5	61	VTQTTA	0.173
		6	27	PEMRGE	0.157
		7	81	ITSGSC	0.151
		8	87	ARPTSA	0.138
		9	32	EEPSTT	0.124
		10	48	TTGSSS	0.112
		11	79	PAITSG	0.103
		12	22	MEPEHP	0.093
		13	49	TGSSSF	0.090
		14	39	WSGGGG	0.085
		15	24	PEHPEM	0.071
		16	43	GGTRST	0.067
		17	40	SGGGGT	0.062
		18	47	STTGSS	0.062
		19	16	PLVEKL	0.062
		20	72	KEGSCP	0.060
		21	21	LMEPEH	0.060
		22	69	ERVKEG	0.055
		23	36	TTKWSG	0.053
		24	83	SGSCAR	0.049
		25	42	GGGTRS	0.049
		26	17	LVEKLM	0.048

		27	100	PSGCRW	0.036
		28	68	RERVKE	0.036
		29	14	FPPLVE	0.022
		30	41	GGGGTR	0.017
		31	82	TSGSCA	0.016
		32	12	LFFPPL	0.014
		33	97	SKRPSG	0.013
		34	30	RGEEPS	0.012
		35	104	RWTTSS	0.001
		36	77	PRPAIT	-0.006
		37	75	SCPRPA	-0.008
		38	23	EPEHPE	-0.008
		39	46	RSTTGS	-0.009
		40	9	TCPLFF	-0.010
		41	80	AITSGS	-0.011
		42	73	EGSCPR	-0.011
		43	29	MRGEEP	-0.014
		44	45	TRSTTG	-0.021
		45	96	PSKRPS	-0.022
		46	15	PPLVEK	-0.022
		47	38	KWSGGG	-0.024
		48	64	TTARRE	-0.024
		49	52	SSFRKS	-0.025
		50	34	PSTTKW	-0.027
VCP	DGMSTKKMCSSLALPTGLTSLILPSSDLAVLISSSTSHFLMRSPVLPSSRLT CASPQLPRMWTWSSWLK	1	27	PSSDLA	5.771
		2	48	VLPSSR	0.312
		3	25	ILPSSD	0.279
		4	33	VLISSS	0.259
		5	19	TGLTSL	0.158
		6	24	LILPSS	0.152
		7	35	ISSSTS	0.138
		8	16	ALPTGL	0.123
		9	47	PVLPSS	0.115
		10	32	AVLISS	0.114
		11	31	LAVLIS	0.113
		12	34	LISSST	0.110
		13	21	LTSLIL	0.109
		14	15	LALPTG	0.096
		15	18	PTGLTS	0.089
		16	29	SDLAVL	0.087
		17	12	CSSLAL	0.086
		18	26	LPSSDL	0.067
		19	36	SSSTSH	0.065

		20	64	MWTWSS	0.062
		21	54	LTCASP	0.054
		22	49	LPSSRL	0.039
		23	44	MRSPVL	0.026
		24	38	STSHFL	0.024
		25	57	ASPQLP	0.022
		26	50	PSSRLT	0.015
		27	9	KKMCSS	0.010
		28	56	CASPQL	-0.004
		29	10	KMCSSL	-0.004
		30	53	RLTCAS	-0.009
		31	28	SSDLAV	-0.009
		32	45	RSPVLP	-0.017
		33	67	WSSWLK	-0.029
		34	42	FLMRSP	-0.029
		35	30	DLAVLI	-0.030
		36	11	MCSSLA	-0.035
		37	17	LPTGLT	-0.038
		38	13	SSLALP	-0.039
		39	46	SPVLPS	-0.044
		40	14	SLALPT	-0.046
		41	66	TWSSWL	-0.047
		42	60	QLPRMW	-0.052
		43	22	TSLILP	-0.052
		44	65	WTWSSW	-0.054
		45	23	SLILPS	-0.063
		46	52	SRLTCA	-0.074
		47	6	MSTKKM	-0.078
		48	37	SSTSHF	-0.081
		49	20	GLTSLI	-0.081
		50	62	PRMWTW	-0.084
XYLT2	DFHLYGSYPP ARQPSRPTGR TPTTRLMAPV GSVMSCSLLT QPSPASACT	1	36	VMSCSL	0.340
		2	33	VGSVMS	0.268
		3	45	PSPASA	0.211
		4	68	LSPGAC	0.137

		5	61	WAPHSA	0.130
		6	65	SAGLSP	0.116
		7	29	LMAPVG	0.093
		8	30	MAPVGS	0.091
		9	42	LTQPSP	0.087
		10	34	GSVMSC	0.077
		11	63	PHSAGL	0.050
		12	31	APVGSV	0.049
		13	57	LHPQWA	0.046
		14	66	AGLSPG	0.045
		15	80	ISMTTI	0.039
		16	52	TMPPLL	0.035
		17	41	LLTQPS	0.028
		18	70	PGACRP	0.026
		19	85	ISRATW	0.024
		20	67	GLSPGA	0.016
		21	7	LYGSYP	0.016
		22	82	MTTISR	0.015
		23	20	PTGRTP	0.014
		24	64	HSAGLS	0.014
		25	14	ARQPSR	0.009
		26	32	PVGSVM	0.008
		27	48	ASACTM	0.003
		28	17	PSRPTG	-0.002
		29	10	SYPPAR	-0.017
		30	18	SRPTGR	-0.018
		31	47	PASACT	-0.020
		32	37	MSCSLL	-0.020
		33	6	HLYGSY	-0.025
		34	76	ACTCIS	-0.028
		35	44	QPSPAS	-0.032
		36	43	TQPSPA	-0.037
		37	5	FHLYGS	-0.040
		38	62	APHSAG	-0.043
		39	53	MPPLLH	-0.045
		40	84	TISRAT	-0.046
		41	8	YGSYPP	-0.052
		42	56	LLHPQW	-0.053
		43	55	PLLHPQ	-0.054
		44	38	SCSLLT	-0.055
		45	39	CSLLTQ	-0.057
		46	13	PARQPS	-0.060
		47	46	SPASAC	-0.061

		48	25	PTTRLM	-0.061
		49	27	TRLMAP	-0.063
		50	77	CTCISM	-0.065
ZBTB20	SASNGTPLQAHPQVPALAPQAWWPARRGPLTSAPSAQQSLTKSSSSTTT	1	17	VPALAP	0.196
		2	34	TSAPSA	0.123
		3	20	LAPQAW	0.116
		4	38	SAQQSL	0.116
		5	43	LTKSSS	0.106
		6	15	PQVPAL	0.093
		7	42	SLTKSS	0.081
		8	30	RGPLTS	0.073
		9	33	LTSAPS	0.057
		10	44	TKSSSS	0.051
		11	37	PSAQQS	0.050
		12	35	SAPSAQ	0.044
		13	45	KSSSST	0.041
		14	8	GTPLQA	0.024
		15	31	GPLTSA	0.023
		16	19	ALAPQA	0.016
		17	6	SNGTPL	0.012
		18	16	QVPALA	0.009
		19	11	LQAHPQ	-0.005
		20	32	PLTSAP	-0.014
		21	41	QSLTKS	-0.022
		22	13	AHPQVP	-0.029
		23	47	SSSTTT	-0.031
		24	18	PALAPQ	-0.032
		25	22	PQAWWP	-0.035
		26	4	SASNGT	-0.036
		27	46	SSSSTT	-0.038
		28	7	NGTPLQ	-0.052
		29	27	PARRGP	-0.052
		30	26	WPARRG	-0.061
		31	10	PLQAHP	-0.070
		32	29	RRGPLT	-0.079
		33	28	ARRGPL	-0.080
		34	40	QQSLTK	-0.085
		35	36	APSAQQ	-0.085
		36	23	QAWWPA	-0.088
		37	39	AQQSLT	-0.090
		38	5	ASNGTP	-0.092
		39	24	AWWPAR	-0.096

		40	25	WWPARR	-0.097
		41	9	TPLQAH	-0.109
		42	21	APQAWW	-0.121
		43	14	HPQVPA	-0.122
		44	12	QAHPQV	-0.144
